# Supplementary figures and images for: A non-neutralizing antibody broadly protects against influenza virus infection by engaging effector cells
Source: PLoS Pathog. 2021 Aug 5;17(8):e1009724. doi: 10.1371/journal.ppat.1009724 (PMC8341508; doi:10.1371/journal.ppat.1009724)

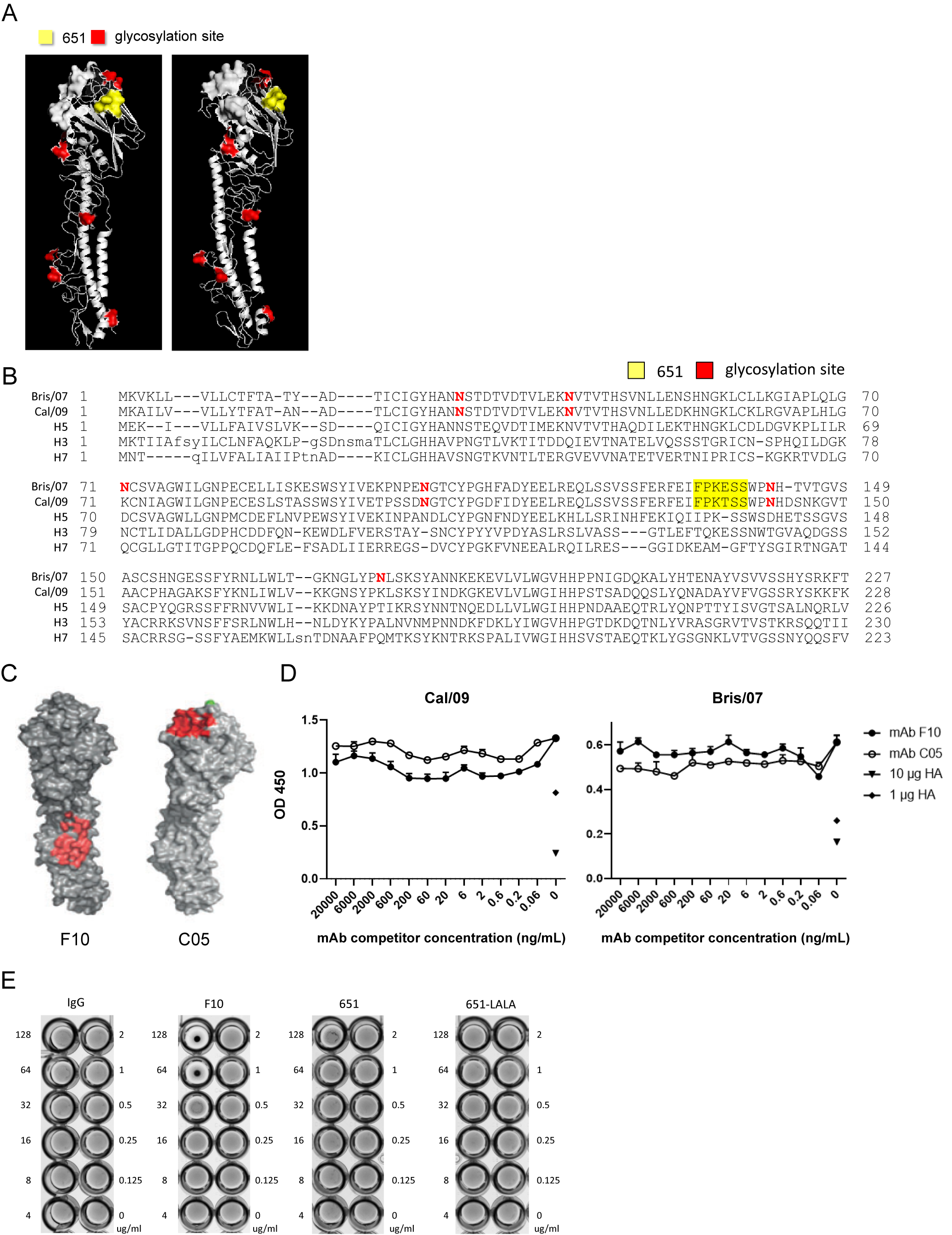

Supplement: S1 Fig — (A) HDX-MS analysis map of the Bris/07 HA-binding sites of mAb 651 (yellow). N-glycosylation sites are marked in red. (B) Multiple sequence alignment of HA amino acid sequences of indicated influenza virus strains using CLUSTALW (website: https://www.genome.jp/tools-bin/clustalw). mAb-bound amino acids mapped by HDX-MS are highlighted in yellow, and N-glycosylation sites on Bris/07 and Cal/09 are labeled in red. (C) The binding epitopes of mAb F10 (left) and mAb C05 (right) on HA are labeled in red. (D) Competitive ELISA showing various concentrations of mAb F10 or mAb C05 did not compete the binding of 651 with Cal/09 HA (left panel) or Bris/07 HA (right panel). Effective competition of 651 binding by full length Cal/09 (left panel) or Bris/07 HA (right panel) at two different doses was also indicated. Results are mean ± SEM (n = 3 for the F10 and C05 groups). (E) mAb 651 did not provide hemagglutination inhibition (HI) activity. The HI titers of Cal/09 virus were determined by incubation of 0.2% turkey red blood cells with indicated mAb at 2-fold serial dilutions. F10 and IgG served as the positive control and negative control for HI activity, respectively. (TIF) [file ppat.1009724.s001.tif]

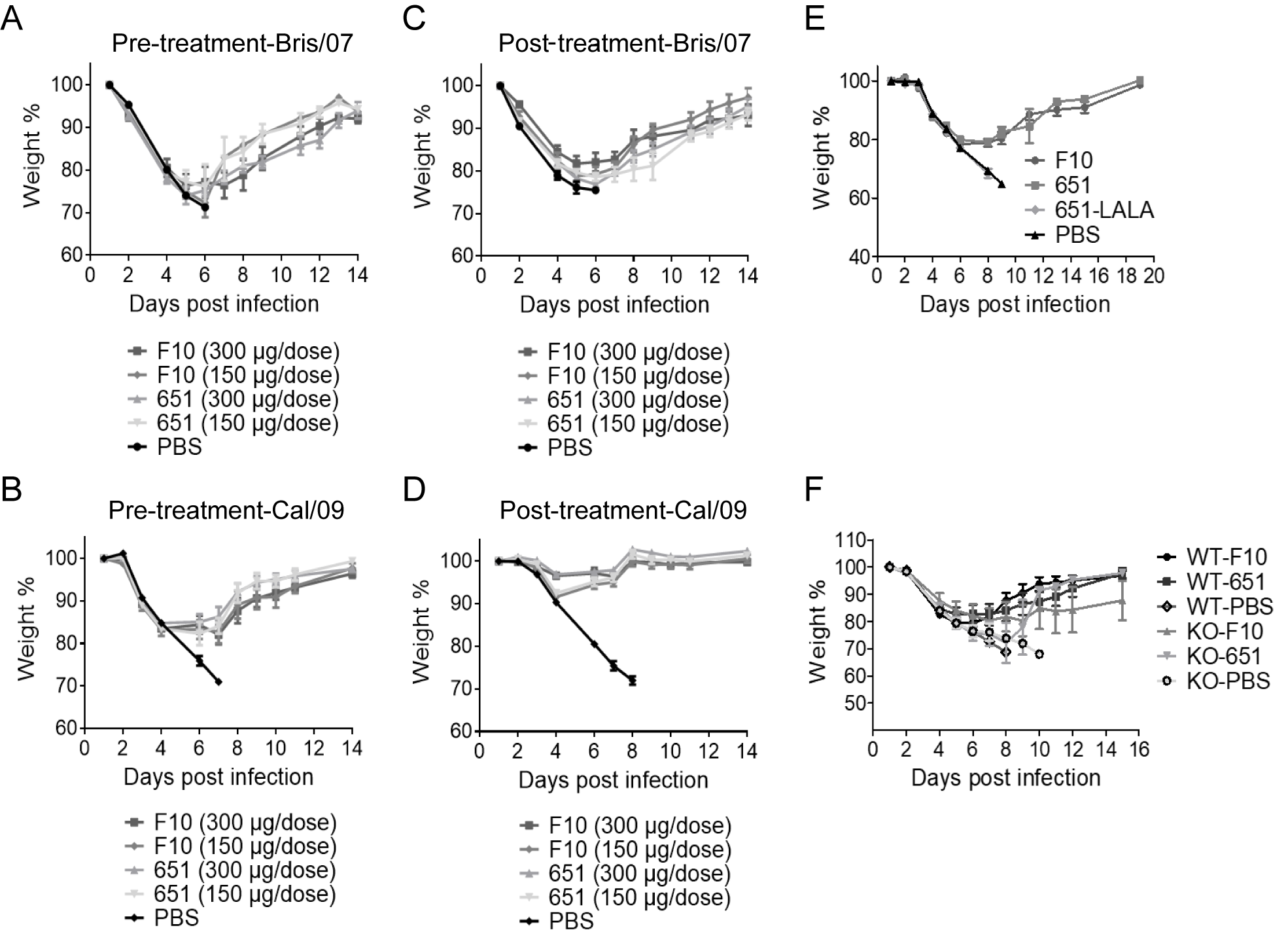

Supplement: S2 Fig — (A and B) Mice were pretreated (i.p.) with indicated mAbs 2 h before intranasal challenge with 100 LD50 of H1N1 Bri/07 virus (A) or Cal/09 virus (B). Body weights were monitored daily for 14 days. n = 5–8 mice per group. (C and D) Mice were i.p. pretreated with indicated mAbs 24 h after intranasal challenge with 100 LD50 of H1N1 Bri/07 virus (C) or Cal/09 virus (D). Body weights were monitored daily for 14 days. n = 4–8 mice per group. (E) Unlike 651, 651-LALA-pretreatment did not prevent weight loss caused by Cal/09 infection. n = 8 mice per group. (F) Weight changes in FcγR knockout (KO) and control WT mice were observed after F10 or 651 pretreatment and Cal/09 infection. F10 mAb was used as the positive control. n = 6–8 mice per group. (TIF) [file ppat.1009724.s002.tif]

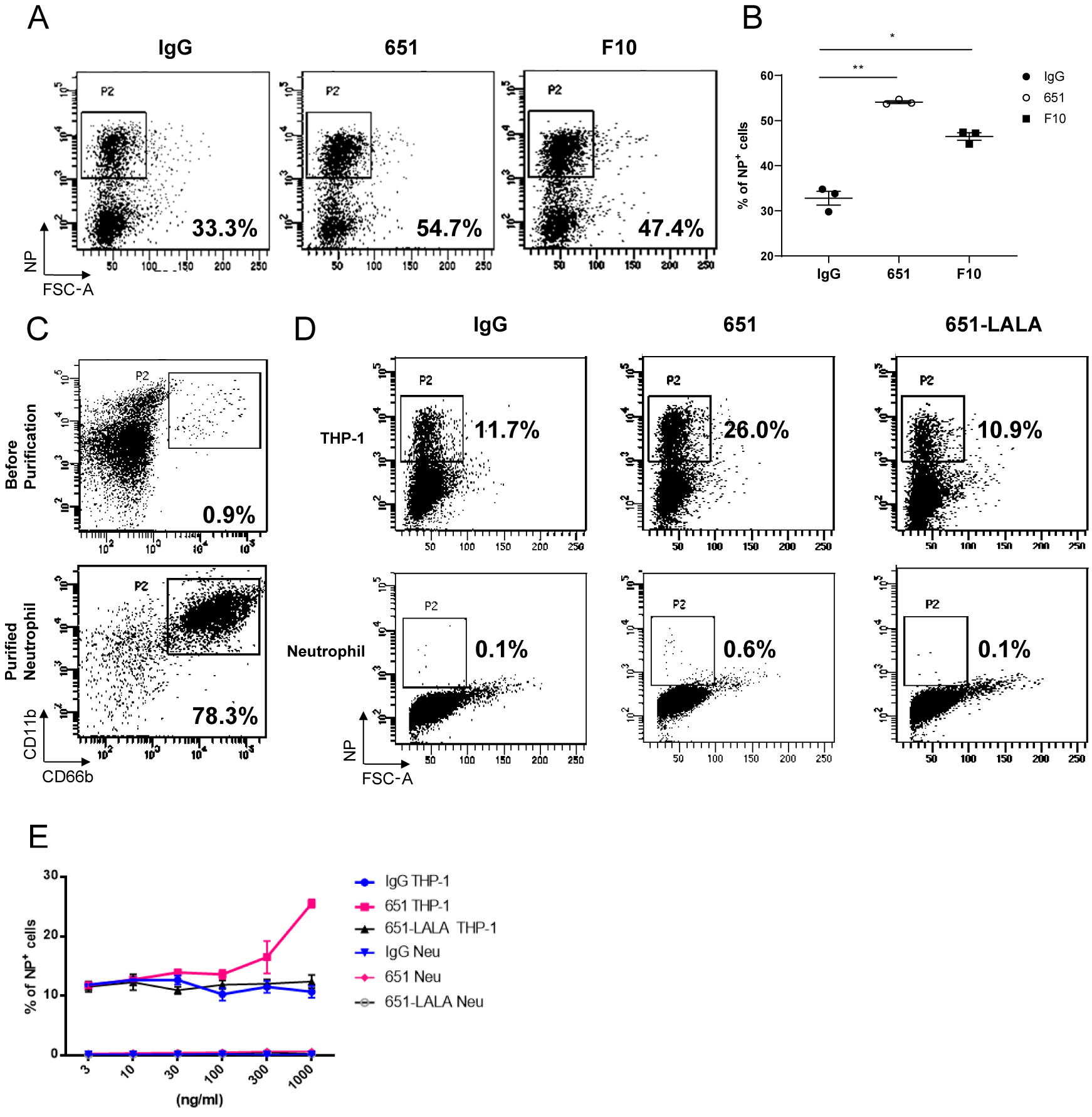

Supplement: S3 Fig — (A) FACS showing the NP positive signals in sialidase (0.5 unit/mL) treated THP-1 cells incubated with 651, F10, or IgG (all at 3,000 ng/mL) opsonized Cal/09 virus. Percentage of NP positive cells is indicated. (B) FACS results showing the percentage of NP positive signals in sialidase treated THP-1 cells incubated with serial doses of 651, F10 or IgG opsonized Cal/09 virus. (C) FACS showing the purity of neutrophils (CD11b+CD66b+) isolated from human peripheral blood by using dextran sedimentation and Ficoll-Hypaque density gradient centrifugation. (D) FACS showing the NP positive signals in sialidase (0.5 unit/mL) treated THP-1 cells (upper panel) and neutrophils from human peripheral blood (lower panel) incubated with 651-, 651-LALA-, or IgG- (all at 1,000 ng/mL) opsonized Cal/09 virus. Percentage of NP positive cells is indicated. (E) FACS results showing the percentage of NP positive signals in sialidase treated THP-1 cells and human neutrophils (Neu) incubated with serial doses of 651, 651-LALA mAbs or IgG opsonized Cal/09 virus. Results are mean ± SEM (n = 3 in B and E). (TIF) [file ppat.1009724.s003.tif]

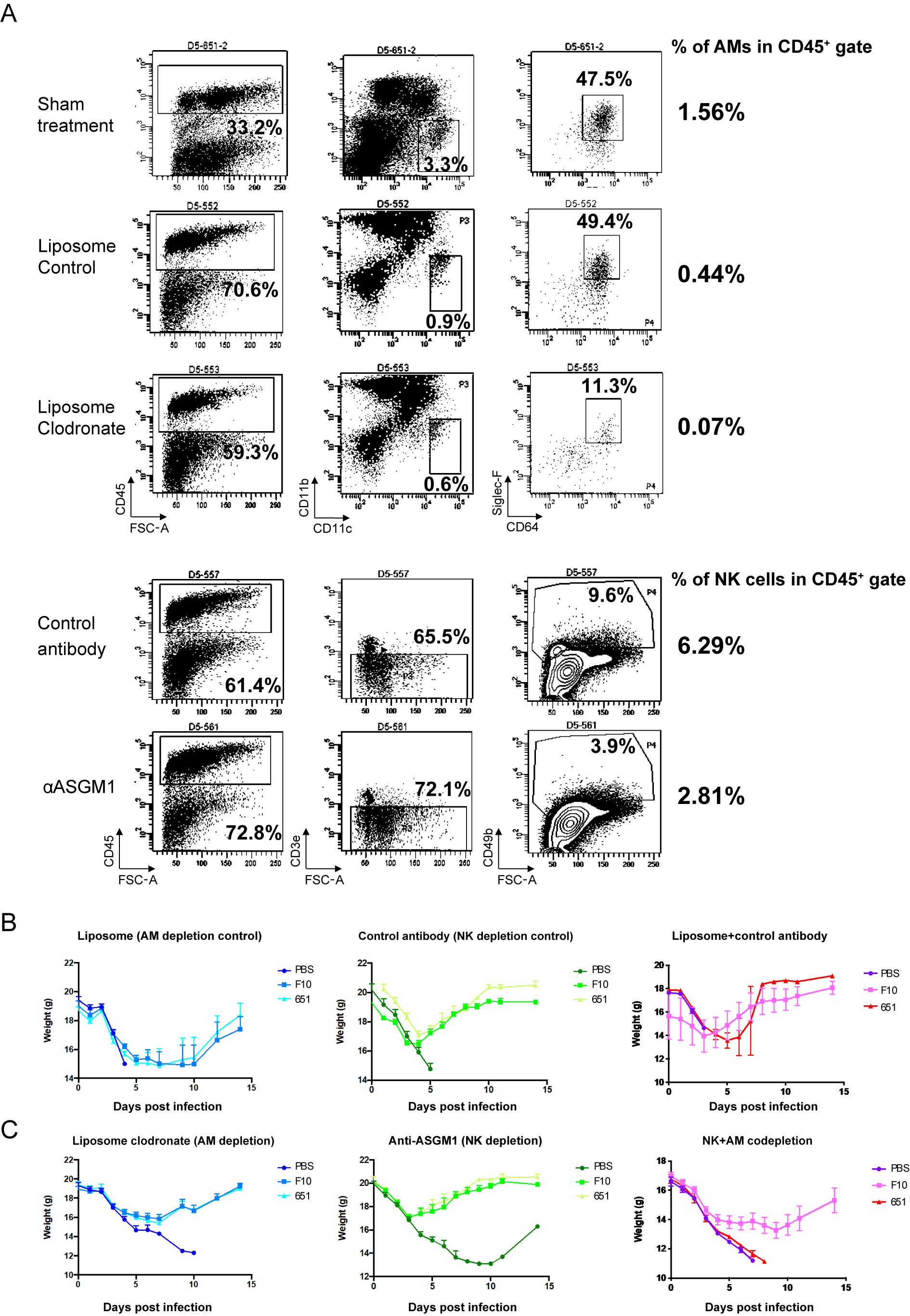

Supplement: S4 Fig — (A) Flow cytometric analysis showing a significant reduction in the frequency of alveolar macrophages (AMs, SiglecF+CD11c+CD11b− CD64+) or NK cells (CD49b+CD3e−) in lungs on day 5 after employing the control or depletion reagents and infection. (B and C) Weight changes of mice treated with control reagents (B) or depleted with AMs and NK cells (C) after pretreatment of 651 (300 μg/dose), F10 (300 μg/dose), or PBS for 2 h before intranasal challenge with 100 LD50 of H1N1 Cal/09 virus. Results are mean ± SEM. n = 8–9 mice per group in B and C. (TIF) [file ppat.1009724.s004.tif]
