# Supplementary material for: A non-neutralizing antibody broadly protects against influenza virus infection by engaging effector cells
Source: PLoS Pathog. 2021 Aug 5;17(8):e1009724. doi: 10.1371/journal.ppat.1009724 (PMC8341508; doi:10.1371/journal.ppat.1009724)
Supplement: Attachment — Submitted filename: PPATHOGENS-D-20-02567_reviewer_comments.pdf [file ppat.1009724.s005.pdf]

PLOS Pathogens

A non-neutralizing antibody broadly protects against influenza virus infection by engaging effector cells

--Manuscript Draft--

**Manuscript Number:**	PPATHOGENS-D-20-02567
**Full Title:**	A non-neutralizing antibody broadly protects against influenza virus infection by engaging effector cells
**Short Title:**	Broadly protective antibody against Flu
**Article Type:**	Research Article
**Section/Category:**	Virology
**Keywords:**	influenza virus; monoclonal antibody; effector function
**Abstract:**	Hemagglutinin (HA) is the immunodominant protein of the influenza virus. We previously showed that mice injected with a monoglycosylated influenza A HA (HA mg) produced cross-strain-reactive antibodies and were better protected than mice injected with a fully glycosylated HA (HA fg) during lethal dose challenge. We employed a single B-cell screening platform to isolate the cross-protective monoclonal antibody (mAb) 651 from mice immunized with the HA mg of A/ Brisbane/59/2007 (H1N1) influenza virus (Bris/07). The mAb 651 recognized the head domain of a broad spectrum of HAs from groups 1 and 2 influenza A viruses and offered prophylactic and therapeutic efficacy against A/California/07/2009 (H1N1) (Cal/09) and Bris/07 infections in mice. The antibody did not possess neutralizing activity; however, antibody-dependent cellular cytotoxicity and antibody-dependent cellular phagocytosis mediated by natural killer cells and alveolar macrophages were important in the protective efficacy of mAb 651. Together, this study highlighted the significance of effector functions for non-neutralizing antibodies to exhibit protection against influenza virus infection.
**Additional Information:**	
**Question**	**Response**
**Financial Disclosure**<br><br>Enter a financial disclosure statement that describes the sources of funding for the work included in this submission. Review the submission guidelines for detailed requirements. View published research articles from *PLOS Pathogens* for specific examples.<br><br>This statement is required for submission and **will appear in the published article** if the submission is accepted. Please make sure it is accurate.	This work was supported by grants from Academia Sinica (AS-SUMMIT-109 to CHW, AS-IA-107-L05 to KIL), Taiwan, and Ministry of Science and Technology (MOST 109-2320-B-001-023-MY3 to KIL).<br>The funders had no role in study design, data collection and analysis, decision to publish, or preparation of the manuscript.

**Unfunded studies**<br>Enter: *The author(s) received no specific funding for this work.*<br><br>**Funded studies**<br><br>Enter a statement with the following details:<br>• Initials of the authors who received each award<br>• Grant numbers awarded to each author<br>• The full name of each funder<br>• URL of each funder website<br>• Did the sponsors or funders play any role in the study design, data collection and analysis, decision to publish, or preparation of the manuscript?<br>• **NO** - Include this sentence at the end of your statement: *The funders had no role in study design, data collection and analysis, decision to publish, or preparation of the manuscript.*<br>• **YES** - Specify the role(s) played.<br><br>* typeset	
**Competing Interests**<br><br>Use the instructions below to enter a competing interest statement for this submission. On behalf of all authors, disclose any competing interests that could be perceived to bias this work—acknowledging all financial support and any other relevant financial or non-financial competing interests.<br><br>This statement **will appear in the published article** if the submission is accepted. Please make sure it is accurate. View published research articles from *PLOS Pathogens* for specific examples.	The authors have declared that no conflicts of interest exist.

**NO authors have competing interests**<br><br>Enter: *The authors have declared that no competing interests exist.*<br><br>**Authors with competing interests**<br><br>Enter competing interest details beginning with this statement:<br><br>*I have read the journal's policy and the authors of this manuscript have the following competing interests: [insert competing interests here]*<br><br>* typeset	
**Data Availability**<br><br>Authors are required to make all data underlying the findings described fully available, without restriction, and from the time of publication. PLOS allows rare exceptions to address legal and ethical concerns. See the PLOS Data Policy and FAQ for detailed information.<br><br>A Data Availability Statement describing where the data can be found is required at submission. Your answers to this question constitute the Data Availability Statement and **will be published in the article**, if accepted.<br><br>**Important:** Stating 'data available on request from the author' is not sufficient. If your data are only available upon request, select 'No' for the first question and explain your exceptional situation in the text box.<br><br>Do the authors confirm that all data underlying the findings described in their manuscript are fully available without restriction?	Yes - all data are fully available without restriction
**Describe where the data may be found in full sentences. If you are copying our sample text, replace any instances of XXX with the appropriate details.**<br>• If the data are **held or will be held in a**	NA

**public repository**, include URLs, accession numbers or DOIs. If this information will only be available after acceptance, indicate this by ticking the box below. For example: *All XXX files are available from the XXX database (accession number(s) XXX, XXX.).*<br>• If the data are all contained **within the manuscript and/or Supporting Information files**, enter the following: *All relevant data are within the manuscript and its Supporting Information files.*<br>• If neither of these applies but you are able to provide **details of access elsewhere**, with or without limitations, please do so. For example:<br><br>*Data cannot be shared publicly because of [XXX]. Data are available from the XXX Institutional Data Access / Ethics Committee (contact via XXX) for researchers who meet the criteria for access to confidential data.*<br><br>*The data underlying the results presented in the study are available from (include the name of the third party and contact information or URL).*<br>• This text is appropriate if the data are owned by a third party and authors do not have permission to share the data.<br><br>* typeset	
Additional data availability information:	

Powered by Editorial Manager® and ProduXion Manager® from Aries Systems Corporation

manuscript text Click here to access/download;Manuscript;Ko et al Text Plos pathogens 1129.pdf

A non-neutralizing antibody broadly protects against influenza virus infection by engaging effector cells

Yi-An Ko[1, #], Yueh-Hsiang Yu[1, #], Yen-Fei Wu[1], Yung-Chieh Tseng[1], Chia-Lin Chen[1], King-Siang Goh[1], Hsin-Yu Liao[1], Ting-Hua Chen[1], Ting-Jen Rachel Cheng[1], An-Suei Yang[1], Chi-Huey Wong[1], Che Ma[1, 2] and Kuo-I Lin[1, 2, *]

[1]Genomics Research Center, Academia Sinica, Taipei 11529, Taiwan

[#] Equal contribution

[2]C. M. and K.-I L. share senior authorship

Address correspondence and reprint requests to

Kuo-I Lin, Genomics Research Center, Academia Sinica, No. 128, Section 2, Academia Road, Nankang District, Taipei 115, Taiwan. Phone: +886-2-2787-1253, Fax: +886-2-2789-9931; email: kuoilin@gate.sinica.edu.tw

Left Running head: Ko et al.

Right Running head: Broadly protective antibody against Flu

Abstract word count: 159

Abstract

Hemagglutinin (HA) is the immunodominant protein of the influenza virus. We previously showed that mice injected with a monoglycosylated influenza A HA (HA_{mg}) produced cross-strain-reactive antibodies and were better protected than mice injected with a fully glycosylated HA (HA_{fg}) during lethal dose challenge. We employed a single B-cell screening platform to isolate the cross-protective monoclonal antibody (mAb) 651 from mice immunized with the HA_{mg} of A/ Brisbane/59/2007 (H1N1) influenza virus (Bris/07). The mAb 651 recognized the head domain of a broad spectrum of HAs from groups 1 and 2 influenza A viruses and offered prophylactic and therapeutic efficacy against A/California/07/2009 (H1N1) (Cal/09) and Bris/07 infections in mice. The antibody did not possess neutralizing activity; however, antibody-dependent cellular cytotoxicity and antibody-dependent cellular phagocytosis mediated by nature killer cells and alveolar macrophages were important in the protective efficacy of mAb 651. Together, this study highlighted the significance of effector functions for non-neutralizing antibodies to exhibit protection against influenza virus infection.

Author Summary

The protective efficacy of antibodies is generally related to their neutralization potency. Here, we isolated a monoclonal antibody from mice injected with monoglycosylated hemagglutinin

protein-based universal flu vaccine, and demonstrated a head-domain recognizing, but non-neutralizing, monoclonal antibody carried prophylactic and therapeutic efficacy against a broad spectrum of influenza virus infections *in vivo* via effector functions.

Introduction

Influenza viral infections cause a contagious respiratory illness of the upper airways and lungs. Approximately half a million deaths worldwide are due to seasonal flu each year [1]. Influenza viruses belong to the *Orthomyxoviridae* family, whose genomes encode 11 proteins, including the major surface glycoproteins hemagglutinin (HA) and neuraminidase (NA) [2]. Influenza viruses are classified into three subtypes: A, B, and C. Among which, the A and B subtypes are responsible for the seasonal flu in humans [2]. Although the influenza vaccine is currently in use for controlling the spread of seasonal flu each year, the trivalent inactivated influenza vaccine only confers protection in around two thirds of those vaccinated [3]. More severe complications resulting from influenza viral infection and a loss of influenza vaccine efficacy have been found in older adults [4, 5]. Thus, there remains a need to develop new strategies to overcome the moderate responses and narrow coverage range of the current flu vaccine.

Advances in antibody engineering technologies have enabled the isolation of several neutralizing antibodies against influenza viruses from either infected patients or donors with extensive vaccinations [6-10]. These anti-flu neutralizing monoclonal antibodies (mAbs) target HA, which comprises a variable immunodominant globular head domain or a more conserved immune-subdominant stem domain [11, 12]. Most of the broadly neutralizing antibodies target the stem region of HA and have less potent direct neutralization activity [11,

12]. Other neutralizing antibodies bind to the head region of HA and generally possess a potent ability to directly inhibit virus entry [12]. Recently, non-neutralizing mAbs recognizing the HA globular head domain trimer interface have been reported that limit the spread of influenza viruses and protect against infection by various flu strains in mice [10, 13]. In addition to the abovementioned modes of action, antibodies can eliminate infected cells through effector functions, such as antibody-dependent cellular cytotoxicity (ADCC) [14], in which nature killer (NK) cells with Fc receptors (FcRs) are the primary effector cells [15]. It has been demonstrated that mAbs targeting the stem region of HA can mediate ADCC [16]. Additionally, antibody-dependent cellular phagocytosis (ADCP), which involves the ingestion of antibody-opsonized influenza virus particles by alveolar macrophages, has also been shown to be involved in the clearance of influenza viruses [17].

Changing the glycan abundance of HA can affect its immunogenicity [18, 19]. We developed a monoglycosylated HA (HA_{mg}) protein-based universal flu vaccine by treating HA with endoglycosidase H. HA_{mg} served as an effective vaccine providing broader protection against infection by various influenza virus strains than the fully glycosylated HA (HA_{fg}) in animal models [20, 21]. One mAb, 651, isolated from HA_{mg}-immunized mice, was subjected to functional characterization in this study. Although it lacks neutralizing activities *in vitro*, 651 is able to recognize the head region of a broad range of HA proteins and protect against infection by several H1N1 viruses through effector functions *in vivo*. Thus, we demonstrated a

unique mode of action for a non-neutralizing mAb that can offer cross-strain protection against influenza virus infection through FcR-mediated effector functions. The results highlight the potential significance of non-neutralizing antibodies in host responses against viral infections.

Results and Discussion

Generation of mAb 651 from Bris/07 HA_{mg}-immunized mice

We previously demonstrated that the HA_{mg} vaccine elicits antibody responses that recognize a broader spectrum of influenza viruses than the HA_{fg} vaccine [21]. We aimed to isolate broadly neutralizing mAbs from mice immunized with HA_{mg} proteins prepared from the A/Brisbane/59/2007 (H1N1) (Bris/07) influenza virus. By using a single B-cell antibody screening technique, we isolated and expressed the HA_{mg}-specific chimeric mAbs (Fig 1A). We identified on a specific mAb, 651, which recognized the broad recognition of HA proteins from different strains of influenza virus, including group 1 and group 2 influenza A virus subtypes (Fig 1B). Quantitative measurement using an antibody affinity assay showed that 651 possessed a higher affinity to HA_{mg} than HA_{fg} from both A/Brisbane/59/2007 (H1N1) (Bris/07) and A/California/7/09 (H1N1) (Cal/09) (Fig 1C).

The structure and conformation of HA are important for its recognition by 651, as decreased binding was observed after HA was treated with 2-ME, which disrupted its protein

conformation (Fig 1D). ELISA results showed that 651 bound to the intact HA, but not to the stem region, of Bris/07 (Fig 1E), implying that 651 mainly recognizes the globular head domain of HA. Supporting this notion, the results of a hydrogen-deuterium exchange-mass spectrometry (HDX-MS) assay showed that there was one region recognized by 651. The mAb 651 interacts with the globular head region near a glycosylation site of HA from Bris/07 (S1A Fig). Sequence alignment of the identified Bris/07 HA peptide with the HA proteins from several influenza viruses revealed that this 651-binding region in the head globular domain is relatively conserved in Bris/07 and Cal/09, and is close to the N-glycosylation site (S1B Fig). Typically, anti-influenza virus antibodies that recognize the globular head domain possess hemagglutination inhibition (HI) and neutralizing activity but show less cross-reactivity compared with antibodies against the stem region [12, 22]. Although 651 recognizes the HA of a panel of influenza viruses, it does not possess neutralizing activity against Bris/07 (H1), Cal/09 (H1), NIBRG-14 (H5), or Vic/11 (H3) influenza viruses (Fig 1F). Furthermore, 651 did not show HI activity against the Cal/09 virus (S1C Fig). Therefore, we have isolated a mAb 651 derived from Bris/07 HA_{mg}-immunized mice that targets the globular head region of various HA proteins but has no neutralizing activity.

mAb 651 confers preventive and therapeutic efficacy through FcγR

Because 651 binds to various strains of influenza virus, we next sought to understand its biological functions *in vivo*. Mice were intraperitoneally injected with two different 651 doses 2 h before viral infection (Fig 2A). To our surprise, pretreatment with 651 provided a dose-dependent protection against Bris/07 infection (Fig 2B) and a complete protection against Cal/09 infection (Fig 2C). The better prophylactic efficacy against Cal/09 correlated with the stronger binding affinity of 651 to the HA of the Cal/09 strain than to that of Bris/07 (Fig 1C).

We then examined the therapeutic efficacy of 651. Mice were administered the antibodies 1 day after infection with influenza virus (Fig 2D). Again, the survival rates were significantly improved in the 651-treated groups after Cal/09 infection. Improved, although not statistically significant, survival rates were observed in the high-dose 651-treated mice after Bris/07 infection (Fig 2E). Remarkably, mice provided a high dose of 651 1 day after Cal/09 infection were completely protected from a lethal dose Cal/09 challenge on day 14 (Fig 2F). Weight loss following viral infection was also prevented in the mice treated with 651 before or after infection (S2A–S2D Fig). F10 was used as a positive control antibody, as it has prophylactic and therapeutic efficacy against a broad spectrum of group 1 human influenza A viruses [23]. Our results showed 651 and F10 had comparable preventive and therapeutic efficacies (Fig 2A–2F and S2A–S2D Fig). Thus, 651 provided notable protective efficacy against influenza viral infection *in vivo*, despite being a non-neutralizing antibody.

In addition to being able to directly neutralize pathogens, antibodies can eliminate infection through FcR-mediated reactions, such as ADCC or ADCP [12]. Because 651 was unable to neutralize the influenza virus, we next examined whether the protective efficacy of 651 was due to FcR-mediated effector functions. We generated an Fc-region mutant with leucine 234 and 235 to alanine substitutions, called 651-LALA, which mitigates antibody effector function by abolishing antibody binding to the FcR [24]. We found that mice given 651-LALA had similar mortality rates as those pretreated with PBS (Fig 2G), demonstrating the significance of the effector function of 651. The γ chain of the Fc receptor is a critical component of the high-affinity receptor for IgG [15]. Mice lacking the FcR γ chain displayed impaired NK-cell-mediated ADCC and macrophage-mediated ADCP [25]. Significantly reduced survival rates were also found in 651-pretreated *Fcer1g*-knockout mice compared with 651-pretreated wildtype mice after Cal/09 infection (Fig 2H). However, a lack of *Fcer1g* did not abolish the protective effect of F10, indicating that neutralizing activity predominantly contributed to the function of F10 (Fig 2H). Following challenge with a lethal dose of Cal/09 virus, mice pretreated with 651-LALA or *Fcer1g*-knockout mice consistently showed similar dramatic bodyweight loss as the PBS treated group (S2E and S2F Fig). Together these results indicate that the protective effects mediated by 651 rely on FcR-mediated effector functions.

651-pretreatment alleviates virus replication and ameliorates lung inflammation

Upon influenza viral infection, both virus-induced virulence and immunopathology contribute to inflammation and tissue injury in the respiratory tract [26]. We next examined whether 651 leads to viral clearance and alleviates lung inflammation. Virus replication was abolished in mice pretreated with F10 2 h before Cal09 infection by 3 days post infection, which is likely to be due to the potent influenza-virus-neutralizing ability of F10 (Fig 3A). Notably, a significant improvement in viral clearance was seen on day 5, but not day 3, in Cal09-infected mice pretreated with 651 (Fig 3A). Hematoxylin and eosin (H&E) staining further showed the reduced pulmonary edema and immune cell infiltration in mice pretreated with F10 or 651. However, mice treated with 651-LALA displayed lung damage and immune cell infiltration as severe as that seen in PBS-treated mice on day 3 after Cal09 infection (Fig 3B).

The viral nucleic acids released upon influenza virus infection can be sensed by various pattern-recognition receptors, which trigger robust downstream signaling [27, 28]. Several cytokines in the lungs, including the type I IFN, MCP-1, IL-6, and TNFα produced by various cells after influenza infection, lead to cytokine storms—one of the major causes of severe flu-associated complications [27]. We next assessed whether the production of cytokines in the lungs is influenced by 651 administration. We found that the production of cytokines, including IFNα, IFNβ, MCP-1, IL-6, and TNFα, was elevated on days 3 and 5 after Cal/09 infection (Fig 3D and 3E). Pretreatment with F10 or 651 significantly reduced the release of all cytokines on day 3 (Fig 3D). At 5 days post infection, the production of IFNβ, MCP-1, and

IL-6 remained significantly reduced by pretreatment with F10 or 651 (Fig 3E). Type III IFN, IFNλ, has been shown to have therapeutic efficacy against the influenza virus, but it does not induce proinflammatory side effects [29]. It is noteworthy that the IFNλ level was elevated in mice pretreated with F10 or 651 on day 3 post infection (Fig 3D). The 651-mediated reduction of cytokine production was reverted when Leu234/Leu235 sites were mutated (Fig 3D and 3E). Therefore, similarly to pretreatment with F10, pretreatment with 651 alleviated lung inflammation after influenza virus challenge.

ADCC and ADCP mediated by 651

The HA of influenza virus was reported to be the antigenic determinant responsible for the generation of antibodies with ADCC [30]. Several immune cell types, including NK cells and neutrophils, target antibody-labeled cells after infection and release cytotoxic granules and cytokines to kill the virus-infected cells [31]. Following Cal/09 challenge, the percentage of NK cells, defined as $CD49b^{+}CD3e^{-}$, in the lungs increased on day 3 (Fig 4A) but then declined to a level similar to that in uninfected mice on day 5 (Fig 4B). We found an increased NK cell population in the lungs on day 5 post infection in F10- and 651-, but not in 651-LALA-, pretreated mice (Fig 4A and 4B), suggesting the potential role of NK cells in mice provided with protective antibody treatment. Furthermore, we used HEK293T cells to express HA proteins from various strains of influenza virus, including Bri/09 H1N1, Cal/09

H1N1, H3N2, and H7N9, to examine whether 651 provided ADCC *in vitro*. Compared with the effects in the control IgG-treated cells, both 651 and FI6 showed greater dose-dependent cytotoxicity towards cells expressing Bris/07, Cal/09, and H3 (Fig 4C), but 651 did not provide ADCC against H7-expressing cells (Fig 4C). This is consistent with our data showing the lack of binding to H7 by 651 (Fig 1B). Since ADCC relies on the Fc-FcR interaction, 651-LALA could not trigger effective ADCC against Bris/07 and Cal/09 HA (Fig 4D).

Besides ADCC, ADCP also contributes to protection against influenza virus infection. Non-neutralizing antibodies have been shown to offer protective effects through alveolar macrophages but not NK cells [32]. We found that on days 3 and 5 after Cal/09 challenge, the frequency of alveolar macrophages significantly declined (Fig 4E and 4F), which is consistent with a previous report showing that alveolar macrophages dramatically diminished in BLAB/c mice after influenza virus infection [33]. Pretreatment with F10 or 651 significantly rescued the decline of alveolar macrophages caused by influenza virus infection on days 3 and 5 post infection (Fig 4E and 4F). We subsequently used THP-1 as effector cells to confirm whether 651 utilizes ADCP for influenza virus clearance *in vitro*. The antibody opsonized Cal/09 virus was incubated with sialidase-treated THP-1 cells, and intracellular viral N protein (NP) was detected. We found that the uptake of viruses by THP-1 cells was accelerated by opsonized 651 (Fig 4G) compared with uptake by the IgG- and 651-LALA-opsonized group. Moreover, a dose-dependent ADCP by 651 was observed (Fig

4H). ADCP activity was not found for various doses of 651-LALA opsonized with Cal/09 (Fig 4G and 4H). Therefore, we demonstrated that 651 possesses ADCC and ADCP activity *in vitro*.

NK cells and alveolar macrophages coordinate the antiviral responses mediated by 651

Having demonstrated the ADCC and ADCP mediated by 651 *in vitro*, we next examined the involvement of NK cells and alveolar macrophages in 651-mediated protection during virus challenge *in vivo*. Mice were administered an anti-ASGM1 antibody to deplete NK cells (S3A Fig) and intranasally administered clodronate liposomes to deplete alveolar macrophages (S3A Fig). As expected, both F10 and 651 still possessed prophylactic activity in Cal/09-infected mice injected with the control antibody or liposomes, as shown by the significantly improved survival (Fig 5A). Remarkably, the prophylactic effects of 651 diminished when NK cells and alveolar macrophages were co-depleted (Fig 5B), while 651 still protected mice lacking either NK cells or alveolar macrophages. In contrast, F10-administered mice remained resistant to influenza virus challenge, even when NK cells and alveolar macrophages were co-depleted (Fig 5B), implying the predominant neutralizing activity of F10 [23]. The body weight loss results demonstrated that the co-depletion of NK cells and alveolar macrophages was required to eradicate the protective effect of 651 (S3B and S3C Fig). Consistently, the production of IFN-β and proinflammatory cytokines, such as

IL-6 and MCP-1, in the lungs was comparable between the 651- and PBS-pretreated mice co-depleted with NK cells and alveolar macrophages on day 3 (Fig 5C) and day 5 (Fig 5D) post infection. The viral titers on day 5 post infection were also similar between the PBS- and 651-pretreated mice co-depleted with NK cells and alveolar macrophages (Fig 5E). These results suggest that the protective efficacy of 651 *in vivo* could be attributed to both NK cells and alveolar macrophages.

From our study, we concluded that 651 is a non-neutralizing mAb with a broad spectrum of recognition range that is able to alleviate the inflammatory responses and ameliorate the mortality associated with influenza viral infection. This broad-spectrum mAb was isolated from HA_{mg}-immunized mice. It remains to be ascertained if a natural influenza viral infection or influenza vaccination in humans can elicit a significant abundance of such broad-recognition and non-neutralizing anti-HA antibodies that confer protection through effector functions. One recent study demonstrated the antibody-dependent enhancement of influenza virus infection involved the recognition of the head domain and the promotion of viral fusion [34]. Studying the structure of HA, 651, and the FcR tertiary complex will further reveal the allosteric preferences of engaging target cells and effector cells for effective ADCC. Nevertheless, the Fab of 651 recognizes the globular head region, near the glycan modification sites of HA, whose sequences are conserved in Bris/07 and Cal/09. We consider that removal or truncation of the glycan may allow the exposure of hidden epitopes of HA

that are relatively conserved across influenza viruses.

Accumulating efforts have improved the potency of antibody-mediated effector functions by generating Fc variants or modulating the glycan compositions of Fc [35-37]. Further modifications of 651 may optimize its protection efficacy *in vivo*. We found that, in addition to NK-cell-mediated ADCC, alveolar macrophage-mediated ADCP is also critical to the protective effects of 651. In response to viral infections, alveolar macrophages are activated, becoming phagocytic and producing large quantities of inflammatory cytokines, including type I IFNs [38]. Some mAbs have been demonstrated to confer protection against influenza viral infection through the action of antibody-induced inflammation and ADCP mediated by alveolar macrophages [32]. However, neither F10 nor 651 increased the production of inflammatory cytokines in the lungs during influenza virus infection, possibly because of the differential cytokine kinetics resulting from the different doses and types of virus used. Although awaiting confirmation in future studies, it is also plausible that the antibody affinity in this study selectively modulated certain subsets of the heterogeneous alveolar macrophages, which are, at minimum, composed of resident macrophages that populate the lungs during embryogenesis and circulating blood monocytes recruited to the lungs following infection [39].

In conclusion, the study described herein revealed the protective effects of a broad-recognition and non-neutralizing mAb against influenza infection, which may prove

valuable in future assessments of vaccine efficacy and therapeutic antibody development.

Materials and Methods

Influenza viruses, HA expression plasmids and HA proteins

The vaccine strains of influenza viruses A/H1N1/California/07/2009 (Cal/09), A/H1N1/Brisbane/59/2007 (Bris/07), A/H5N1/Vietnam/1194/2004/NIBRG14 (NIBRG14) and A/H3N2/Victoria/361/2011 (Vic/11) were from the reference collection of the National Institute for Biological Standards and Control. All viruses were inoculated into the allantoic cavities of 10-d-old specific pathogen-free embryonated chicken eggs for 2 days at 35°C. The 50% tissue culture infective dose ($TCID_{50}$) of viruses in Madin-Darby canine kidney (MDCK) (CCL-34; American Type Culture Collection) cells and LD_{50} of virus in BALB/c mice were determined before experiments. Methods for expression and purification of the ectodomains of HA from Cal/09, Bris/07, NIBRG14, Vic/11, A/H1N1/WSN/1933 (WSN), A/H1N1/Puerto Rico/8/1934 (PR8), A/H7N9/Shanghai/2/2013, and B/Victoria/2/87 were described previously [20]. Methods for generation and purification of stem region of HA from Bris/07 as well as HA_{mg} and HA_{fg} from Cal/09 and Bris/07 were described previously [21, 40].

Flow cytometry

To determine the immune cell subsets in lungs of infected mice, whole lung tissues were

removed, followed by isolation of single cell suspensions using the lung dissociation kit (Miltenyi Biotec). Cells were harvested and suspended in FACS buffer (2% FBS in PBS) at a density of 10^6/ml. The antibodies used in this study are anti-mouse B220 antibody (BD, clone RA3-6B2), anti-mouse IgG1 antibody (BD, clone X56), anti-mouse CD38 antibody (Biolegend, clone 90), anti-mouse CD45 antibody (BD, clone 30-F11), anti-mouse CD49b antibody (Biolegend, clone DX5), anti-mouse CD3e antibody (BD, clone 145-2c11), anti-mouse Siglec-F antibody (BD, clone E50-2440), anti-mouse CD64 antibody (BD, clone X54-5/7.1), anti-mouse CD11b antibody (BD, clone M1/70), and anti-mouse CD11c antibody (Biolegend, clone N418). Cellular fluorescence intensity was analyzed by FACSCanto (BD Biosciences) and FCS Express 3.0 software.

Cloning and expression of HA-specific mAbs

Ig genes from a single B cell were isolated primarily following the protocols reported previously [21, 41, 42]. Briefly, single cell was collected from the spleens of BALB/c mice immunized i.m. two times at 2 weeks apart with 20 μg Bris/07 HA_{fg} or HA_{mg}. The $HA_{fg}^{+}B220^{+}IgG1^{+}CD38^{+}$ splenic B cells were isolated on day 29 by a cell sorter (FACSAria II), and the single B cell was sorted into 96-well PCR plates (Thermal Scientific). RT-PCR reactions were performed as described previously [21, 41, 42]. Aliquots of nested PCR products were sequenced and analyzed using IMGT/V-Quest (http://www.imgt.org) to

identify the highest homology gene loci of germ-line V, D, and J genes. Those candidate Ig heavy- and light-chain cDNA segments were further subcloned to a chimeric Ig expression vector modified from the tandem chimeric antibody expression (TCAE) vector and the pIgG1(κ) vector (provided by Dr. T. W. Chang, Genomics Research Center, Academia Sinica, Taipei, Taiwan). The generation of the mutations at Fc region of mAb 651, Leu234Ala/Leu235Ala (LALA), was performed by substituting two leucine (L) residues with alanine (A) at a.a. 234 and 235 through site-directed mutagenesis of cDNA. The positive control of anti-HA antibodies FI6 [6] and F10 [23] were expressed and purified following previously described procedures [43].

Purification and binding efficacy of recombinant mAbs

Ig expression vector was transfected into HEK293F cells by using Expi293^{TM} Expression System Kit (Thermo Fisher Scientific). Three days later, supernatant was collected for antibody purification by using Protein A Sepharose (GE) chromatography. Antibody was dissolved in PBS. Enzyme-linked immunosorbent assay (ELISA) was used to determine the binding of recombinant mAbs with HA. Briefly, purified HA of Cal/09, Bris/07, WSN/33, PR8/34, H3, H5, H7 and Flu B viruses was coated on the 96-well plates (0.1 μg/well in 100 μL) for 2 h. The HA-coated plates were then incubated with the 2-fold serial dilutions of recombinant mAbs starting from the highest concentration at 0.05 μg/mL for 2 h. The

captured recombinant antibodies were detected by HRP-conjugated anti-human antibodies and peroxidase substrate solution substrate (BD Biosciences). The OD was read by the SpectraMax M2 Microplate Reader (Molecular Devices). The bio-layer interferometry (BLI) was performed on Octet RED 96 instrument (FortéBio, Inc.). Antibody was immobilized onto anti-human AHC biosensors (FortéBio, Inc.) and incubated with HAs at 0.013–3.17 μM for 90 seconds for association and then incubated in 20 mM Tris, pH 8.0, 150 mM NaCl, 0.005% Tween 20 for dissociation for 90 seconds. The signals for each binding event were measured with a 1:1 Langmuir binding model for k_{on}, k_{off} and K_d value determination.

Microneutralization assay

Microneutralization assay was performed as previously described [37]. The freshly prepared virus was quantified with the median TCID50. The 10-fold TCID50 of virus was mixed in equal volume with 2-fold serial dilutions of mAbs in 96-well plates and incubated for 1 h at 37 °C. The mixture was added onto the MDCK cells (1.5×10^4 cells per well) in the plates followed by incubation at 37 °C for 16-20 h. The cells were washed with PBS, fixed in acetone/methanol solution (1:1 vol/vol), and blocked with 5% (wt/vol) skim milk in PBS. Quantification of virus was detected by ELISA with a polyclonal antibody against influenza A nucleoprotein (NP) protein. The anti-NP primary antibody was added and incubated for 1 h at 37 °C. After washing with PBST (PBS + 0.01% Tween 20), the secondary antibody (rabbit

anti-goat IgG HRP conjugated) was added and incubated for 1 h at 37 °C. Peroxidase substrate solution was then added and incubated for 15 min at room temperature in the dark, followed by adding stop solution. The absorbance (OD) of the wells was read at 450/620 nm.

Hemagglutination inhibition assay (HI) assay

The hemagglutination units of Cal/09 virus were determined following a previous report [21]. Briefly, the 2-fold serial dilutions of Cal/09 virus were added into the 96-well round (U) bottom plates, and 0.2% turkey red blood cells (Jianrong Farm, Taiwan) were added into each well and mixed well. After 30 min, the hemagglutination unit (HAU) was identified. For antibody mediated hemagglutination inhibition detection, 1024 HAU of Cal/09 virus was mixed with 2-fold serial dilutions of mAbs and incubated for 30 min. After incubation, 0.2% turkey red blood cells were added into each well and mixed well. After 30 min, the hemagglutination inhibition was determined.

Virus challenge in mice

Female BALB/c mice, purchased from National Laboratory Animal Center, Taiwan, at 8 wk old were injected i.p. with 300 μg (15 mg/kg) or 150 μg (7.5 mg/kg) purified mAb 2 h before or 24 h after intranasal challenge with H1N1 Bris/07 or Cal/09 virus with a lethal dose (100 LD50). In some experiments, FcγR knockout mice (purchased from The Jackson Laboratory)

at 8 wk old were used. Mouse body weight and survival data were measured and recorded every day afterwards. In some experiments, NK cells were depleted by anti-ASGM1 (50 μL/mouse, WAKO) antibody or PBS (as the control) by ip injection 2 d before mAb injection. Macrophages were depleted by clodronate liposomes (100 μL/mouse, ClodronateLiposomes) 2 and 4 d before mAb injection. All animal experiments were evaluated and approved by the Institutional Animal Care and Use Committee of Academia Sinica.

ADCC assay

ADCC was performed as previously reported [37]. HEK293T cells transfected with HA-expression vectors (Bris/07, Cal/09, H3, and H7) for 48 h and human peripheral blood mononuclear cells (PBMCs) were used as target cells and effector cells, respectively. Human PBMCs from healthy donors were obtained from Taipei Blood Center with the consent procedures approved by the Academia Sinica Research Ethics Committee. PBMCs were isolated by density gradient centrifugation with Ficoll-Paque at 400×*g* for 30 min without brake at 22°C. mAbs at 2-fold serial dilutions were added to the co-culture composed of 5×10^3 293T cells transiently expressing HA proteins from indicated strains of influenza viruses and 2.5×10^5 effector PBMCs (E:T ratio= 50), and incubated for 5 h at 37°C. The supernatant of the co-culture was collected and analyzed by CytoTox 96 Non-Radioactive Cytotoxicity Assay Kit (Promega).

ADCP assay

ADCP was performed essentially as a previous report [44]. Briefly, Cal/09 virus was incubated with indicated antibodies at 37 °C for 1 h and added to the sialidase (0.5 unit/mL, Sigma) pre-treated THP-1 cells (from ATCC). After 1 h incubation, THP-1 cells were washed three times with RPMI and incubated at 37 °C. After 6 h incubation, THP-1 cells were fixed in 4% paraformaldehyde, permeabilized and stained with anti-influenza NP antibody (Abcam, Ab20921, 1:100 dilution). The levels of phagocytosis were monitored by flow cytometry.

Histology analysis, cytokine levels and viral titers in lung analysis

The perfused mouse lungs isolated at day 3 and day 5 after infection were fixed in 4% formaldehyde, embedded in paraffin and cut into 3 μm-thick sections for Hematoxylin & Eosin (H&E) staining. To determine the viral titers in the infected lungs, infected mice were sacrificed on day 3 and day 5 post-infection. The whole lungs were collected and homogenized in PBS (2.5 ml/g lung). The viral titers from the homogenates were determined by immunoplaque assay on MDCK cells following previously established protocols [45]. Briefly, MDCK cells were seeded into 24-well plates. One day later, the MDCK monolayers were washed once with PBS. Ten-fold serial dilutions of lung homogenates, made in in serum-free RPMI containing 0.5 μg/mL TPCK-trypsin, were added to the MDCK cells. After

1 h incubation, the cells were washed once with PBS and overlaid with DMEM containing 0.5 μg/mL TPCK-trypsin and 0.5% agarose. After overnight incubation, MDCK cells were fixed with 10% formaldehyde and the agarose were removed. The cells were fixed, permeabilized and stained with anti-influenza NP antibody and the focus forming units were determined. The lung homogenates were used for determining the levels of cytokines by using IFN-β and IFN-α ELISA kits from PBL Assay Science, MCP-1, IL-6, and TNF-α ELISA kits from eBioscience, and IFN-λ2/3 ELISA kit from R&D Systems.

Hydrogen-Deuterium Exchange-Mass Spectrometry (HDX-MS) Assay

HDX-MS was performed essentially as previously reported [46]. Briefly, the Fab regions of the antibodies were prepared by using Pierce™ Fab Preparation Kit (Thermo). HA protein (from Bris/07) and Fab region of antibody were co-incubated for 30 min at 37 °C, digested by PNGase F (NEB) for 2 h at 37 °C. Hydrogen was exchanged to deuterium by adding D_2O to the reaction for 10 and 20 min at room temperature and stopped the exchange by increasing urea concentration to 2 M and low temperature (4°C). After pepsin and protease type XIII digestion, the sample was ready for MALDI TOF analysis with ESI mass spectrometry (Velos Pro LTQ, Thermo Scientific, mass spectrometric core facility, Genomics Research Center, Academia Sinica) to determine the interacting region between HA and Fab.

Statistical analysis

Data are shown as the mean ± SEM. Statistical analyses were performed using GraphPad Prism 8 software. Analysis of differences between two groups was performed by an unpaired two-tailed Student's t test. Comparisons between multiple groups were performed using a one-way ANOVA, followed by Dunnetts' honestly significant difference post hoc test. Survival rate differences between groups were analyzed by using log-rank (Mantel-Cox) test.

Online supplemental material

S1 Fig shows the *in vitro* characterizations of mAb 651-binding to HA.

S2 Fig shows the weight changes of mice pretreated or post-treated with mAbs, followed by influenza virus infection.

S3 Fig shows the depletion efficiency of NK cells and alveolar macrophages (AMs), and the weight changes of mice depleted with AMs and/or NK cells after mAb pretreatment and Cal/09 virus challenge.

Author contributions

K.-I Lin conceived the study. Y.-A. Ko, Y.-H. Yu, Y.-F. Wu, Y.-C. Tseng, C.-L. Chen, K.-S. G., H.-Y. Liao, and T.-H. Chen performed the experiments and data analysis. T.-J. R. Cheng, A.-S. Yang, C.-H. Wong, and C. Ma provided critical reagents and analytic tools. Y.-A. Ko, Y.-H.

Yu and K.-I Lin contributed to writing and revising the manuscript.

Acknowledgments

We thank Shii-Yi Yang and Szu-Teng Ma for excellent technical support. This work was supported by grants from Academia Sinica (AS-SUMMIT-109, AS-IA-107-L05), Taiwan, and Ministry of Science and Technology (MOST 109-2320-B-001-023-MY3).

Conflicts of interest: The authors have declared that no conflicts of interest exist.

References

1. Lambert LC, Fauci AS. Influenza vaccines for the future. N Engl J Med. 2010;363(21):2036-44. doi: 10.1056/NEJMra1002842. PubMed PMID: 21083388.

2. Girard MP, Cherian T, Pervikov Y, Kieny MP. A review of vaccine research and development: human acute respiratory infections. Vaccine. 2005;23(50):5708-24. Epub 2005/09/13. doi: S0264-410X(05)00718-8 [pii]
10.1016/j.vaccine.2005.07.046. PubMed PMID: 16154667.

3. Ostrand-Rosenberg S, Grusby MJ, Clements VK. Cutting edge: STAT6-deficient mice have enhanced tumor immunity to primary and metastatic mammary carcinoma. J Immunol. 2000;165(11):6015-9.

4. McElhaney JE. Influenza vaccine responses in older adults. Ageing research reviews. 2011;10(3):379-88. doi: 10.1016/j.arr.2010.10.008. PubMed PMID: 21055484; PubMed Central PMCID: PMC3061971.

5. Dugan HL, Henry C, Wilson PC. Aging and influenza vaccine-induced immunity. Cell Immunol. 2020;348:103998. doi: 10.1016/j.cellimm.2019.103998. PubMed PMID: 31733824.

6. Corti D, Voss J, Gamblin SJ, Codoni G, Macagno A, Jarrossay D, et al. A neutralizing antibody selected from plasma cells that binds to group 1 and group 2 influenza A hemagglutinins. Science. 2011;333(6044):850-6. doi: 10.1126/science.1205669. PubMed

PMID: 21798894.

7. Ekiert DC, Kashyap AK, Steel J, Rubrum A, Bhabha G, Khayat R, et al. Cross-neutralization of influenza A viruses mediated by a single antibody loop. Nature. 2012;489(7417):526-32. doi: 10.1038/nature11414. PubMed PMID: 22982990; PubMed Central PMCID: PMC3538848.

8. McCarthy KR, Watanabe A, Kuraoka M, Do KT, McGee CE, Sempowski GD, et al. Memory B Cells that Cross-React with Group 1 and Group 2 Influenza A Viruses Are Abundant in Adult Human Repertoires. Immunity. 2018;48(1):174-84 e9. doi: 10.1016/j.immuni.2017.12.009. PubMed PMID: 29343437; PubMed Central PMCID: PMC5810956.

9. Lee J, Boutz DR, Chromikova V, Joyce MG, Vollmers C, Leung K, et al. Molecular-level analysis of the serum antibody repertoire in young adults before and after seasonal influenza vaccination. Nat Med. 2016;22(12):1456-64. doi: 10.1038/nm.4224. PubMed PMID: 27820605; PubMed Central PMCID: PMC5301914.

10. Bangaru S, Lang S, Schotsaert M, Vanderven HA, Zhu X, Kose N, et al. A Site of Vulnerability on the Influenza Virus Hemagglutinin Head Domain Trimer Interface. Cell. 2019;177(5):1136-52 e18. doi: 10.1016/j.cell.2019.04.011. PubMed PMID: 31100268; PubMed Central PMCID: PMC6629437.

11. Murin CD, Wilson IA, Ward AB. Antibody responses to viral infections: a structural

perspective across three different enveloped viruses. Nature microbiology. 2019;4(5):734-47. doi: 10.1038/s41564-019-0392-y. PubMed PMID: 30886356; PubMed Central PMCID: PMC6818971.

12. Krammer F. The human antibody response to influenza A virus infection and vaccination. Nat Rev Immunol. 2019;19(6):383-97. doi: 10.1038/s41577-019-0143-6. PubMed PMID: 30837674.

13. Dong J, Gilchuk I, Li S, Irving R, Goff MT, Turner HL, et al. Anti-influenza H7 human antibody targets antigenic site in hemagglutinin head domain interface. J Clin Invest. 2020. doi: 10.1172/JCI136032. PubMed PMID: 32749241.

14. Von Holle TA, Moody MA. Influenza and Antibody-Dependent Cellular Cytotoxicity. Frontiers in immunology. 2019;10:1457. doi: 10.3389/fimmu.2019.01457. PubMed PMID: 31316510; PubMed Central PMCID: PMC6611398.

15. Nimmerjahn F, Ravetch JV. Fcgamma receptors as regulators of immune responses. Nat Rev Immunol. 2008;8(1):34-47. doi: 10.1038/nri2206. PubMed PMID: 18064051.

16. DiLillo DJ, Tan GS, Palese P, Ravetch JV. Broadly neutralizing hemagglutinin stalk-specific antibodies require FcgammaR interactions for protection against influenza virus in vivo. Nat Med. 2014;20(2):143-51. doi: 10.1038/nm.3443. PubMed PMID: 24412922; PubMed Central PMCID: PMC3966466.

17. Huber VC, Lynch JM, Bucher DJ, Le J, Metzger DW. Fc receptor-mediated phagocytosis

makes a significant contribution to clearance of influenza virus infections. J Immunol. 2001;166(12):7381-8. doi: 10.4049/jimmunol.166.12.7381. PubMed PMID: 11390489.

18. Bajic G, Maron MJ, Adachi Y, Onodera T, McCarthy KR, McGee CE, et al. Influenza Antigen Engineering Focuses Immune Responses to a Subdominant but Broadly Protective Viral Epitope. Cell Host Microbe. 2019;25(6):827-35 e6. doi: 10.1016/j.chom.2019.04.003. PubMed PMID: 31104946; PubMed Central PMCID: PMC6748655.

19. Tseng YC, Wu CY, Liu ML, Chen TH, Chiang WL, Yu YH, et al. Egg-based influenza split virus vaccine with monoglycosylation induces cross-strain protection against influenza virus infections. Proc Natl Acad Sci U S A. 2019;116(10):4200-5. doi: 10.1073/pnas.1819197116. PubMed PMID: 30782805; PubMed Central PMCID: PMC6410868.

20. Wang CC, Chen JR, Tseng YC, Hsu CH, Hung YF, Chen SW, et al. Glycans on influenza hemagglutinin affect receptor binding and immune response. Proc Natl Acad Sci U S A. 2009;106(43):18137-42. Epub 2009/10/14. doi: 0909696106 [pii]
10.1073/pnas.0909696106. PubMed PMID: 19822741; PubMed Central PMCID: PMC2775302.

21. Chen JR, Yu YH, Tseng YC, Chiang WL, Chiang MF, Ko YA, et al. Vaccination of monoglycosylated hemagglutinin induces cross-strain protection against influenza virus infections. Proc Natl Acad Sci U S A. 2014;111(7):2476-81. doi: 10.1073/pnas.1323954111.

PubMed PMID: 24469815; PubMed Central PMCID: PMC3932897.

22. Krause JC, Crowe JE, Jr. Committing the Oldest Sins in the Newest Kind of Ways-Antibodies Targeting the Influenza Virus Type A Hemagglutinin Globular Head. Microbiol Spectr. 2014;2(5). doi: 10.1128/microbiolspec.AID-0021-2014. PubMed PMID: 26104373.

23. Sui J, Hwang WC, Perez S, Wei G, Aird D, Chen LM, et al. Structural and functional bases for broad-spectrum neutralization of avian and human influenza A viruses. Nat Struct Mol Biol. 2009;16(3):265-73. doi: 10.1038/nsmb.1566. PubMed PMID: 19234466; PubMed Central PMCID: PMC2692245.

24. Chappel MS, Isenman DE, Everett M, Xu YY, Dorrington KJ, Klein MH. Identification of the Fc gamma receptor class I binding site in human IgG through the use of recombinant IgG1/IgG2 hybrid and point-mutated antibodies. Proc Natl Acad Sci U S A. 1991;88(20):9036-40. doi: 10.1073/pnas.88.20.9036. PubMed PMID: 1833770; PubMed Central PMCID: PMC52646.

25. Takai T, Li M, Sylvestre D, Clynes R, Ravetch JV. FcR gamma chain deletion results in pleiotrophic effector cell defects. Cell. 1994;76(3):519-29. doi: 10.1016/0092-8674(94)90115-5. PubMed PMID: 8313472.

26. Herold S, Becker C, Ridge KM, Budinger GR. Influenza virus-induced lung injury: pathogenesis and implications for treatment. Eur Respir J. 2015;45(5):1463-78. doi:

10.1183/09031936.00186214. PubMed PMID: 25792631.

27. Guo XJ, Thomas PG. New fronts emerge in the influenza cytokine storm. Semin Immunopathol. 2017;39(5):541-50. doi: 10.1007/s00281-017-0636-y. PubMed PMID: 28555383; PubMed Central PMCID: PMCPMC5580809.

28. Iwasaki A, Pillai PS. Innate immunity to influenza virus infection. Nat Rev Immunol. 2014;14(5):315-28. doi: 10.1038/nri3665. PubMed PMID: 24762827; PubMed Central PMCID: PMCPMC4104278.

29. Davidson S, McCabe TM, Crotta S, Gad HH, Hessel EM, Beinke S, et al. IFNlambda is a potent anti-influenza therapeutic without the inflammatory side effects of IFNalpha treatment. EMBO Mol Med. 2016;8(9):1099-112. doi: 10.15252/emmm.201606413. PubMed PMID: 27520969; PubMed Central PMCID: PMCPMC5009813.

30. Hashimoto G, Wright PF, Karzon DT. Antibody-dependent cell-mediated cytotoxicity against influenza virus-infected cells. J Infect Dis. 1983;148(5):785-94. doi: 10.1093/infdis/148.5.785. PubMed PMID: 6605395.

31. Jegaskanda S, Reading PC, Kent SJ. Influenza-specific antibody-dependent cellular cytotoxicity: toward a universal influenza vaccine. J Immunol. 2014;193(2):469-75. doi: 10.4049/jimmunol.1400432. PubMed PMID: 24994909.

32. He W, Chen CJ, Mullarkey CE, Hamilton JR, Wong CK, Leon PE, et al. Alveolar macrophages are critical for broadly-reactive antibody-mediated protection against influenza

A virus in mice. Nat Commun. 2017;8(1):846. doi: 10.1038/s41467-017-00928-3. PubMed PMID: 29018261; PubMed Central PMCID: PMCPMC5635038.

33. Califano D, Furuya Y, Metzger DW. Effects of Influenza on Alveolar Macrophage Viability Are Dependent on Mouse Genetic Strain. J Immunol. 2018;201(1):134-44. doi: 10.4049/jimmunol.1701406. PubMed PMID: 29760191; PubMed Central PMCID: PMCPMC6008236.

34. Winarski KL, Tang J, Klenow L, Lee J, Coyle EM, Manischewitz J, et al. Antibody-dependent enhancement of influenza disease promoted by increase in hemagglutinin stem flexibility and virus fusion kinetics. Proc Natl Acad Sci U S A. 2019;116(30):15194-9. doi: 10.1073/pnas.1821317116. PubMed PMID: 31296560; PubMed Central PMCID: PMC6660725.

35. Lazar GA, Dang W, Karki S, Vafa O, Peng JS, Hyun L, et al. Engineered antibody Fc variants with enhanced effector function. Proc Natl Acad Sci U S A. 2006;103(11):4005-10. doi: 10.1073/pnas.0508123103. PubMed PMID: 16537476; PubMed Central PMCID: PMC1389705.

36. Li T, DiLillo DJ, Bournazos S, Giddens JP, Ravetch JV, Wang LX. Modulating IgG effector function by Fc glycan engineering. Proc Natl Acad Sci U S A. 2017;114(13):3485-90. doi: 10.1073/pnas.1702173114. PubMed PMID: 28289219; PubMed Central PMCID: PMC5380036.

37. Lin CW, Tsai MH, Li ST, Tsai TI, Chu KC, Liu YC, et al. A common glycan structure on immunoglobulin G for enhancement of effector functions. Proc Natl Acad Sci U S A. 2015;112(34):10611-6. doi: 10.1073/pnas.1513456112. PubMed PMID: 26253764; PubMed Central PMCID: PMC4553773.

38. Divangahi M, King IL, Pernet E. Alveolar macrophages and type I IFN in airway homeostasis and immunity. Trends Immunol. 2015;36(5):307-14. doi: 10.1016/j.it.2015.03.005. PubMed PMID: 25843635.

39. Kopf M, Schneider C, Nobs SP. The development and function of lung-resident macrophages and dendritic cells. Nat Immunol. 2015;16(1):36-44. doi: 10.1038/ni.3052. PubMed PMID: 25521683.

40. Liao HY, Wang SC, Ko YA, Lin KI, Ma C, Cheng TR, et al. Chimeric hemagglutinin vaccine elicits broadly protective CD4 and CD8 T cell responses against multiple influenza strains and subtypes. Proc Natl Acad Sci U S A. 2020;117(30):17757-63. Epub 2020/07/17. doi: 10.1073/pnas.2004783117. PubMed PMID: 32669430; PubMed Central PMCID: PMCPMC7395492.

41. Smith K, Garman L, Wrammert J, Zheng NY, Capra JD, Ahmed R, et al. Rapid generation of fully human monoclonal antibodies specific to a vaccinating antigen. Nature protocols. 2009;4(3):372-84. doi: 10.1038/nprot.2009.3. PubMed PMID: 19247287; PubMed Central PMCID: PMC2750034.

42. Tiller T, Busse CE, Wardemann H. Cloning and expression of murine Ig genes from single B cells. Journal of immunological methods. 2009;350(1-2):183-93. doi: 10.1016/j.jim.2009.08.009. PubMed PMID: 19716372.

43. Chen IC, Chiu YK, Yu CM, Lee CC, Tung CP, Tsou YL, et al. High throughput discovery of influenza virus neutralizing antibodies from phage-displayed synthetic antibody libraries. Scientific reports. 2017;7(1):14455. Epub 2017/11/02. doi: 10.1038/s41598-017-14823-w. PubMed PMID: 29089574; PubMed Central PMCID: PMCPMC5663709.

44. Ana-Sosa-Batiz F, Vanderven H, Jegaskanda S, Johnston A, Rockman S, Laurie K, et al. Influenza-Specific Antibody-Dependent Phagocytosis. PLoS One. 2016;11(4):e0154461. Epub 2016/04/29. doi: 10.1371/journal.pone.0154461. PubMed PMID: 27124730; PubMed Central PMCID: PMCPMC4849649.

45. Tan GS, Krammer F, Eggink D, Kongchanagul A, Moran TM, Palese P. A pan-H1 anti-hemagglutinin monoclonal antibody with potent broad-spectrum efficacy in vivo. J Virol. 2012;86(11):6179-88. Epub 2012/04/12. doi: 10.1128/JVI.00469-12. PubMed PMID: 22491456; PubMed Central PMCID: PMCPMC3372189.

46. Chen HS, Hou SC, Jian JW, Goh KS, Shen ST, Lee YC, et al. Predominant structural configuration of natural antibody repertoires enables potent antibody responses against protein antigens. Scientific reports. 2015;5:12411. Epub 2015/07/24. doi: 10.1038/srep12411.

PubMed PMID: 26202883; PubMed Central PMCID: PMCPMC5378893.

Figure Legends

Figure 1. Generation of 651 from Bris/07 HA_{mg}-immunized mice

(A) Bris/07 (H1N1) HA proteins with two types of glycan modifications, HA_{fg} (HA carrying the typical complex type N-glycans) and HA_{mg} (HA carrying GlcNAc at N-glycosylation sites only), were used to immunize mice, followed by isolation of mAbs with a single B cell screening platform. (B) The resulting recombinant mAb 651 showed cross-recognition of several strains of group A influenza virus. (C) BLI assay showing 651 possessed a higher affinity for HA_{mg} from Cal/09. (D) Immunoblot of reduced (5% 2-ME in sample buffer) and non-reduced Bris/07 HA proteins showing 651 recognized the structural epitope of HA. (E) ELISA showing 651 was unable to recognize the stem region of HA from Bris/07. (F) Effects of 651 on the microneutralization of various strains of influenza viral infections. FI6 and F10 were used as the positive controls.

Figure 2. Prophylactic and therapeutic efficacy of 651 in mice challenged with lethal dose of H1N1

(A-C) Cumulative survival rate of mice treated (i.p.) with indicated mAb 2 h before challenge with 100 LD50 of H1N1 Bris/07 virus (B) or Cal/09 virus (C). (D-F) Cumulative survival rate of mice injected (i.p.) with 651 24 h after challenge with 100 LD50 of H1N1 Bris/07 virus (E) or Cal/09 virus (F). (G) Cumulative survival rate of mice treated with F10, 651, or a mutation

of 651 (651-LALA) after challenge with Cal/09 at 100 LD50. (H) Cumulative survival rate of FcγR knockout (KO) mice and control WT mice after F10 or 651 pretreatment and Cal/09 virus challenge. Data were analyzed by log-rank (Mantel-Cox) test. The statistical significance was calculated for the differences between PBS control and mAb treated groups (in B, C, E, F, and G), or between WT and FcγR knockout (KO) mice receiving the same kind of mAb (in H). Six-8 mice were used per group in each experiment. $^*p < 0.05$; $^{**}p < 0.01$; $^{***}p < 0.001$. N.S. = no significant difference.

Figure 3. Pretreatment with 651 reduced viral replication and alleviated lung inflammation after respiratory challenge with Cal/09 virus

(A and B) Fluorescent focus assay showing the viral titers in lungs of various indicated mAb- or PBS-pretreated mice on day 3 (A) and day 5 (B) after Cal/09 infection. (C) H&E staining showing the histopathology of lungs in mAb-pretreated mice on day 3 after Cal/09 infection. Results from two individual mice from each group are shown. Scale bar = 100 μm. (D and E) Pulmonary IFNα, IFNβ, MCP-1, IL-6, TNFα, and IFNλ2/3 protein levels on day 3 (D) and day 5 (E) after Cal/09 infection were measured by ELISA using samples from mice receiving indicated pretreatments. NC represents mice without infection. Results are mean ± SEM. (n = 4-10). $^*p < 0.05$; $^{**}p < 0.01$; $^{***}p < 0.001$.

Figure 4. 651 possessed ADCC or ADCP against influenza viruses *in vitro*

(A and B) FACS analysis showing the percentage of pulmonary NK cells ($CD49b^{+}$) in $CD45^{+}CD3e^{-}$-gated populations in indicated mAb-pretreated mice on day 3 (A) and day 5 (B) post challenge of Cal09 virus. (C) 651 showed ADCC against H1 (Bris/07 and Cal/09) and H3 but not H7-expressing 293T cells. (D) 651-LALA mutation abolished ADCC mediated by 651 to kill cells expressing H1 from Bris/07 or Cal/09 viruses. IgG was used as the negative control in C and D. (E and F) FACS analysis showing the percentage of alveolar macrophages (AMs) ($SiglecF^{+}CD64^{+}$) in $CD45^{+}CD11b^{-}CD11c^{+}$ gate in indicated mAb- or PBS-pretreated mice on day 3 (E) and day 5 (F) post-challenge with Cal/09 virus. (G) FACS showing the NP positive signals in sialidase- (0.5 unit/mL) treated THP-1 cells incubated with 651-, 651-LALA-, or IgG- (all at 100 ng/ml) opsonized Cal/09 virus. Percentage of NP positive cells is indicated. (H) FACS results showing the percentage of NP positive signals in sialidase treated THP-1 cells incubated with serial doses of 651 or 651-LALA mAbs and Cal/09 virus. Results are mean ± SEM. (n = 3-9). *$p < 0.05$; **$p < 0.01$; ***$p < 0.001$.

Figure 5. 651-mediated protection in mice requires both alveolar macrophages and NK cells

(A and B) Cumulative survival rate of mice receiving control reagents, including liposome alone, control antibody, or both (A), or clodronate liposomes or/and anti-ASGM1 antibody (B)

prior to i.p. injection of mAbs and 100 LD50 Cal/09 intranasal challenge. (C and D) Comparison of pulmonary IFNβ, MCP-1, and IL-6 protein levels measured by ELISA in alveolar macrophages (AM)-NK co-depleted mice treated with either PBS or 651 on day 3 (C) and day 5 (D) post-Cal/09 infection. (E) Fluorescent focus assay showing the comparable influenza virus titers in AM-NK co-depleted mice treated with either PBS or 651 on day 5 after Cal/09 infection. Data in A and B were analyzed by log-rank (Mantel-Cox) test. Data in C-E are mean ± SEM. (n = 4-12). *p < 0.05; **p < 0.01; ***p < 0.001. N.S. = no significant difference.

Figures
Click here to access/download;Figure;Ko et al. Figures.pdf

Ko et al. Figure 1

A

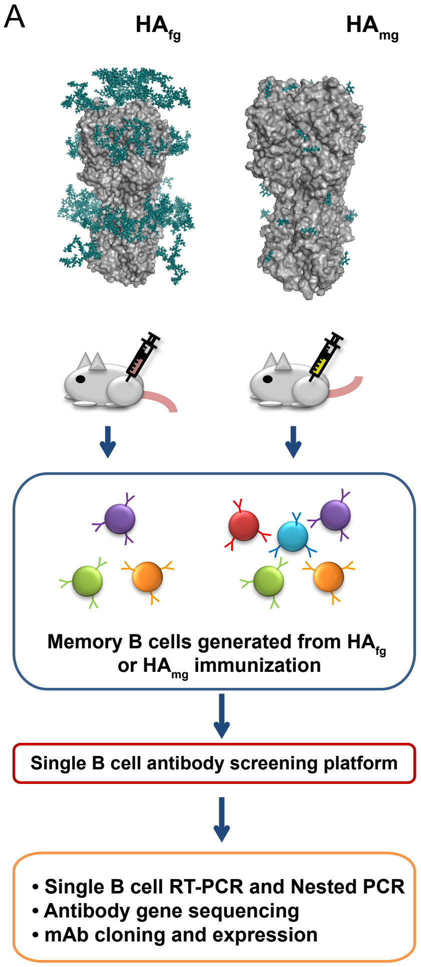

B

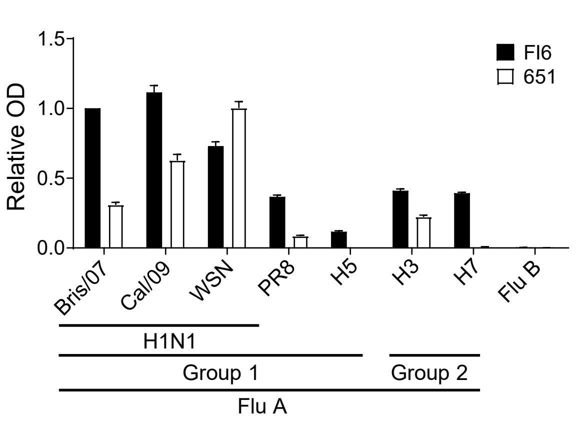

C

		HA$_{fg}$	HA$_{mg}$
Bris/07 HA	K_D (nM)	**96.08 ± 4.26**	**30.19 ± 66.15**
	k_{on} (x10^4 1/Ms)	1.55 ± 0.02	0.64 ± 0.01
	k_{off} (x10^{-3} 1/s)	1.49 ± 0.04	0.19 ± 0.04
Cal/09 HA	K_D (nM)	**0.42 ± 7.28**	**0.20 ± 0.01**
	k_{on} (x10^4 1/Ms)	2.60 ± 0.05	3.35 ± 0.02
	k_{off} (x10^{-5} 1/s)	1.09 ± 0.64	0.69 ± 0.02

D

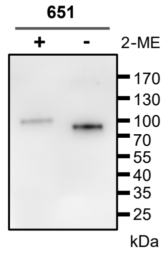

E

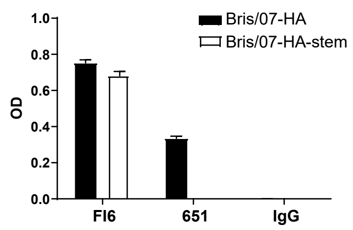

F

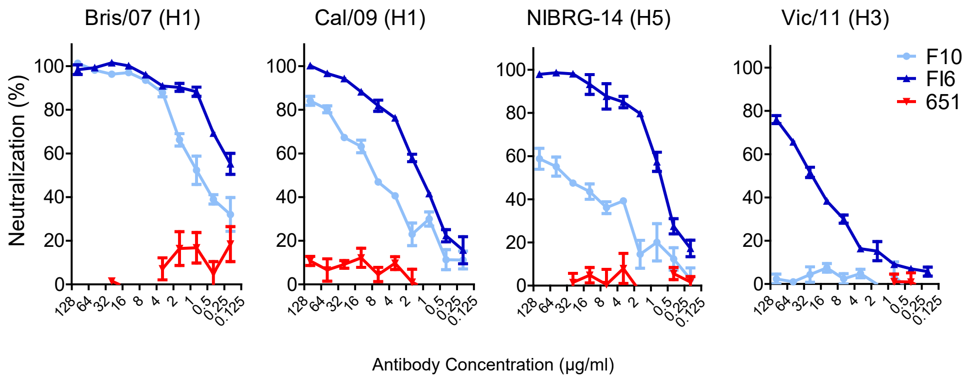

Ko et al. Figure 2

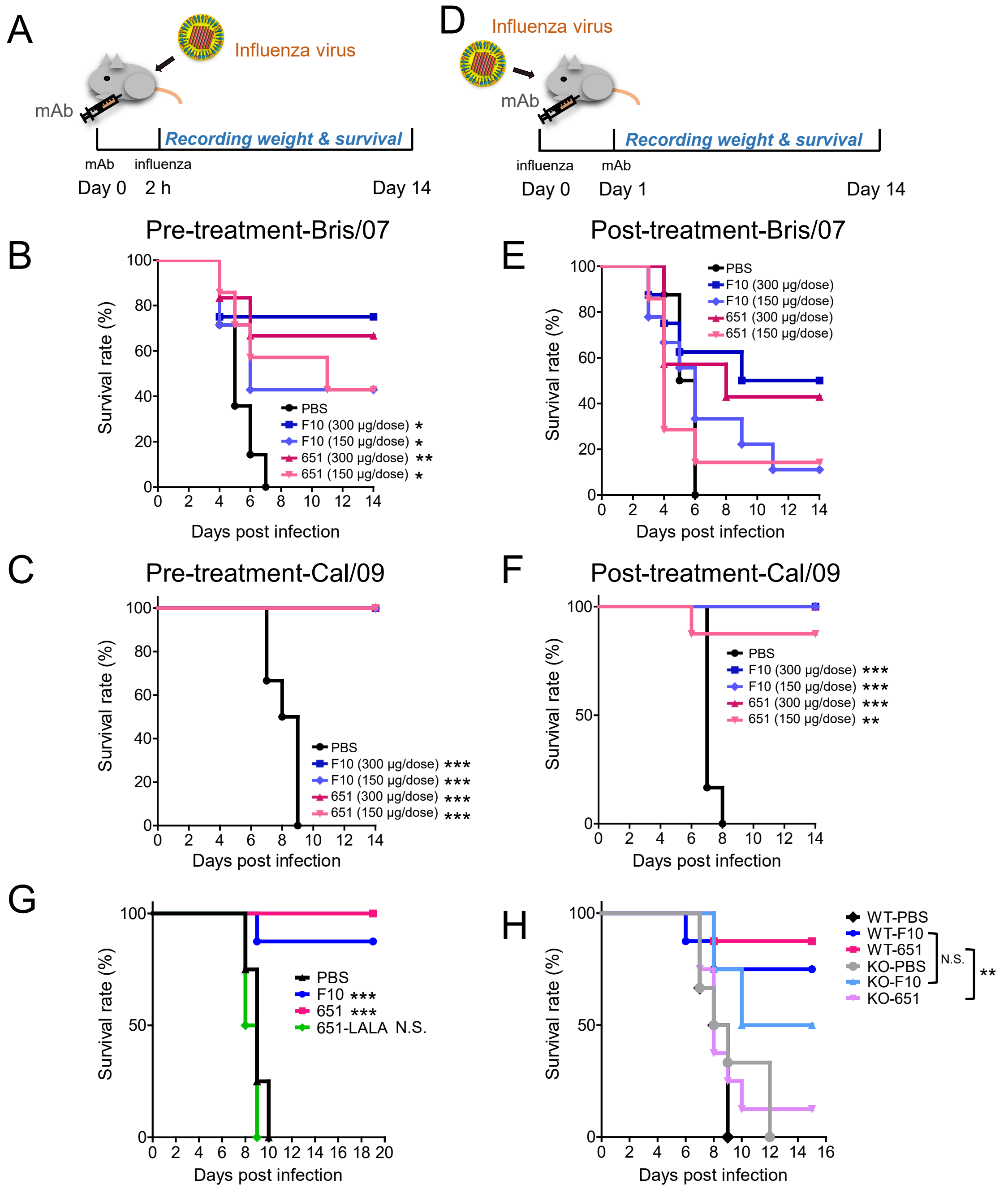

Ko et al. Figure 3

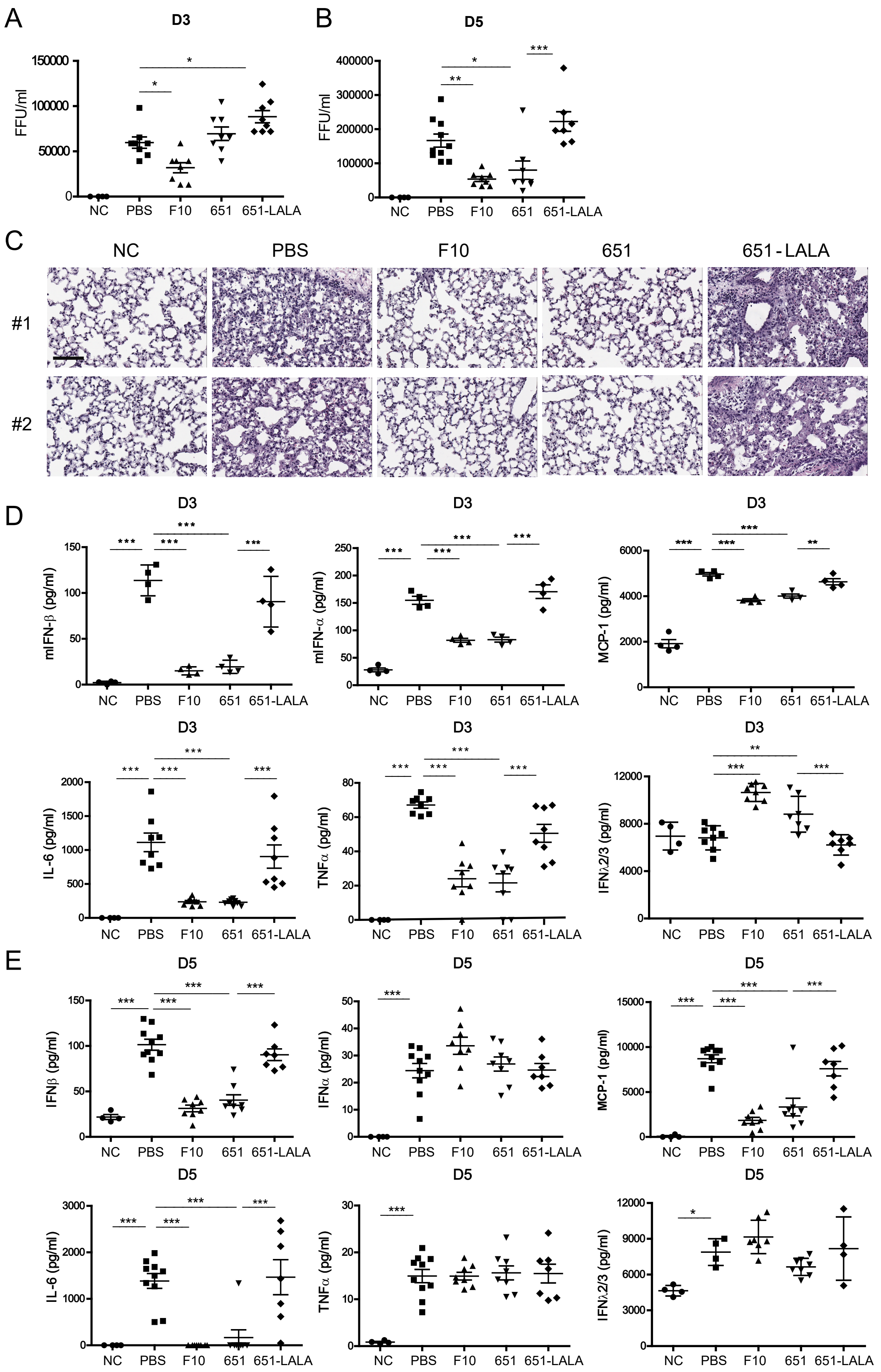

Ko et al. Figure 4

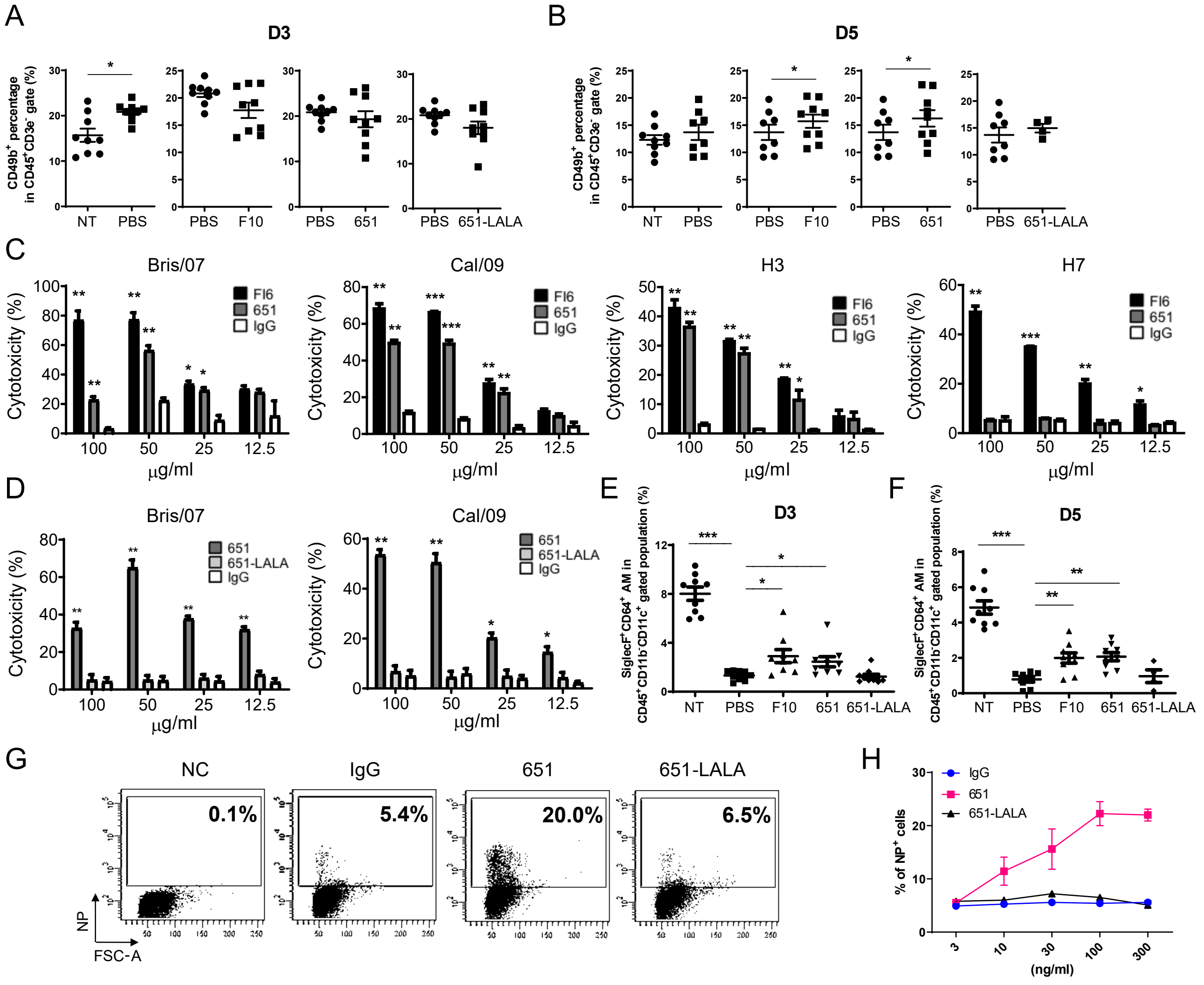

Ko et al. Figure 5

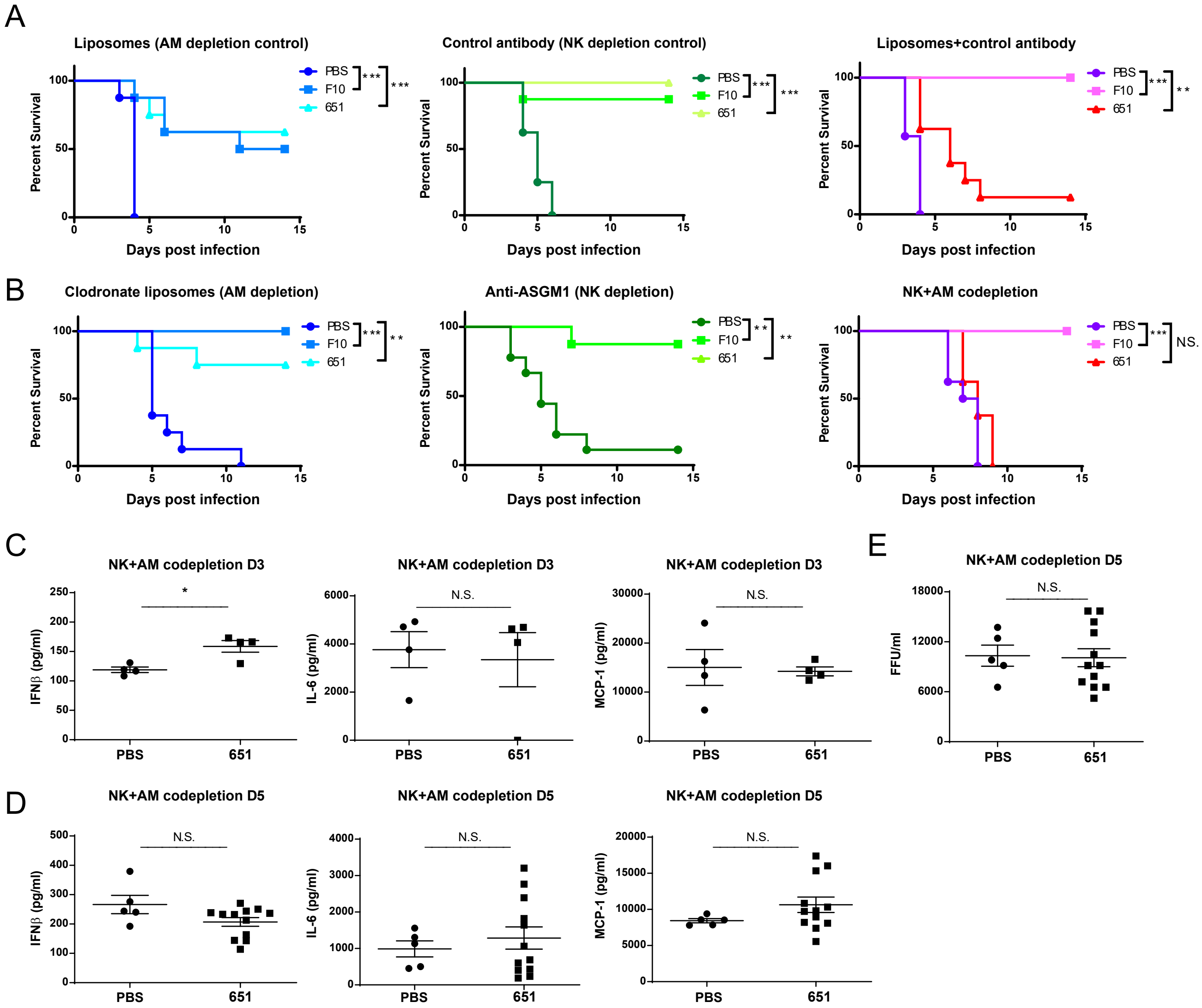

Supplementary figures

Click here to access/download
Supporting Information
Ko et al Supplementary information Plos Pathogens.pdf
