# Supplementary material for: A non-neutralizing antibody broadly protects against influenza virus infection by engaging effector cells
Source: PLoS Pathog. 2021 Aug 5;17(8):e1009724. doi: 10.1371/journal.ppat.1009724 (PMC8341508; doi:10.1371/journal.ppat.1009724)
Supplement: Attachment — Submitted filename: point-by-point reply Plos Pathogens.pdf [file ppat.1009724.s006.pdf]

Reviewer#1

Part I - Summary

Reviewer #1: In general, data presented in this manuscript appear solid and support the conclusion that non-neutralizing antibody is able to have a protective activity against influenza infection via ADCC and ADCP. The authors generated a non-neutralizing antibody, 651, from influenza-infected mice and showed that 651 has little neutralizing activity using in vitro assay. Subsequently, the authors demonstrated that 651 treatment promotes survival of influenza-infected mice and reduces lung inflammation. The authors examined the protective activity of 651 is via ADCC and ADCP, which appear to be dependent on alveolar macrophages and NK cells. Overall, I think the manuscript is acceptable for publication once some corrections and clarifications are addressed.

Reply: We thank the reviewer for the positive comments.

Part II – Major Issues: Key Experiments Required for Acceptance

Reviewer #1: None.

Part III – Minor Issues: Editorial and Data Presentation Modifications

Reviewer #1: See the attachment.

Reply: We thank the reviewer for carefully reading our manuscript and pointing out our errors. We have made the changes accordingly. Our replies were provided in the PDF attached in the "Other information".

The reviewer asked us one additional experiment: *"It is curious how F10 treatment shows NP uptake."* in the ADCP assay. In response to this question, we performed ADCP by THP-1 cells using F10. Our new results shown in the supplementary Figure 3A in this revision indicated that both F10 and 651 accelerated the uptake of influenza virus by THP-1 cells (page 13, line 218).

Reviewer#2

Part I - Summary

Reviewer #2: The manuscript by Ko et al. describes a non-neutralizing antibody (651) that binds to the HA head domain and mediates protection in vivo via the induction of Fc-dependent effector function of alveolar macrophages and NK cells. In general, the study is very well conducted and adds to a growing body of literature demonstrating the importance of non-neutralizing antibodies in protection against influenza virus infection. The only notable weakness is the lack of attention given to neutrophils, a potentially important and abundant Fc-bearing cell type. Other minor comments for the authors' consideration are noted below.

Reply: We thank the reviewer for the positive comments and excellent suggestions. In this revision, we performed additional experiments to address the role of neutrophils in ADCP.

Part II – Major Issues: Key Experiments Required for Acceptance

Reviewer #2: 1. Neutrophils are a highly abundant FcR-expressing leukocyte. They have also been shown to mediate ADCP against influenza virus in vitro (PMID: 27703076) but the importance of their contribution to antibody-mediated protection in vivo is less clear. The authors glaze over the possible contributions of these cells in their manuscript. It would be interesting to perform in vitro ADCP assays using 651 and neutrophils to determine whether they are capable of mediating ADCP. It would also be interesting for the authors to perform a neutrophil depletion experiment in the context of 651 passive transfer (as they did for NK cells and alveolar macrophages) to test the possible contribution of these cells to protection in their system.

Reply: We thank the reviewer for this excellent suggestion. In this revision, we used neutrophils isolated from human peripheral blood to demonstrate that 651 did not accelerate the uptake of influenza virus by neutrophils in vitro. Because of this lack of correlation and depletion of neutrophils may cause other effects independent of antibody effector function in influenza virus infection, we did not further perform the neutrophil deletion experiments. Our new results were shown in the Supplementary Figures 3C~E in this revision (page 13, lines 223-228).

Part III – Minor Issues: Editorial and Data Presentation Modifications

Reviewer #2: 1. Line 44 (and elsewhere): "flu" is too colloquial for scientific publication – please edit to "influenza" Reply: The corrections have been made. Thank you.

2. Line 45: Influenza A viruses are now known to encode up to at least 14 proteins (not 11) – please correct Reply: We have made the correction.

3. Line 47: There are 4 subtypes of influenza virus: A,B,C,D (not 3) – please correct Reply: We have made the correction.

4. Line 60: The lower neutralization capacity of HA stem-binding bnAbs is directly shown in PMID: 25589655 Reply: We have included this reference.

5. Lines 69-71: ADCP mediated by alveolar macrophages has also been shown to have importance in PMID: 29018261 Reply: We have included this reference.

6. Line 72: The authors cite papers showing the impact of reducing glycosylation on antigenicity – it might be worth noting that hyperglycosylation can also influence antigenicity as shown in PMID: 24155380 Reply: We have included this reference. Thank you for the suggestion.

7. Lines 183-184: The authors should note that antibodies against NA have also been reported to elicit ADCC, though less potently than those that bind the HA stem (PMID: 27698132). Reply: We have

included this point and reference in this revision (lines 192-193, page 11). Thank you for the suggestion.

8. The ability of 651 to mediate Fc-dependent effector functions is likely due to its ability to preserve the “two points of contact” previously shown to be essential for HA-specific antibody-mediated ADCC (PMIDs: 27698132, 27647907). This mechanistic explanation would be worth mentioning in the discussion. Reply: We have included this “two points of contact” concept in this revision (lines 266-272, pages 15-16), and cited the reference. Thank you for the suggestion.

Reviewer#3

Part I - Summary

Reviewer #3: This manuscript by Ko et al. describes a broadly cross-reactive 651 mAb that binds to an undefined epitope in the globular head domain of HA and can recognize an array of group 1 & 2 influenza viruses. Using a combination of in vitro assays and mouse work, the authors demonstrate that 651 does not protect mice through virus neutralization but rather through ADCC and ADCP. The novelty of this study is questionable given that this has already been widely reported by many other studies. There are, however, some novel aspects of this study like the NK cell and macrophage co-depletion assays. A major weakness of this study is that there was no attempt made by the authors to define the HA head epitope bound by 651. General execution of this study was sufficient overall, but there were some unexplained results from the co-depletion and cytotoxicity assays that are not satisfactorily addressed.

Reply: We thank the reviewer for noting the novelty of our manuscript. In our original submission, we have performed hydrogen-deuterium exchange-mass spectrometry (HDX-MS) assay to show that one region on the head region, which is distinct from the receptor-binding site, was recognized by 651. In this revision, we performed competitive ELISA to demonstrate that two mAbs, C05 and F10, that recognize the receptor binding site of head region and stem region of HA, respectively, failed to compete the binding of 651 with HA. We have also provided the explanation for our results from the co-depletion and cytotoxicity assays in lines 235~237.

Part II – Major Issues: Key Experiments Required for Acceptance

Reviewer #3: Major concerns:

1. Studies that describe a single novel mAb typically define the exact HA epitope recognized using structural work or at a minimum a competition ELISA. In order for this manuscript to be considered for publication, the exact HA epitope bound by 651 should be established.

Reply: We thank the reviewer for raising this critical point. In our original submission, we performed hydrogen-deuterium exchange-mass spectrometry (HDX-MS) assay to show that one region on the head region (distinct from the receptor-binding site) was recognized by 651. We agreed that the structural study, such as cryo-EM or x-ray crystallography, to fine map the epitope will provide more definite answer of 651 epitope in the level of the atom, but those may be beyond the scope of this study as which primarily focuses on the biological activities of a non-neutralizing antibody in influenza virus infection. To address this point, in this revision, we performed competitive ELISA to demonstrate that two mAbs (C05 and F10, recognizing the receptor-binding site of head region and stem region of HA, respectively), failed to compete the binding of 651 with HA (Supplementary Figures 1C and 1D, page 7, lines 108~113).

2. Why is the 651 mAb not directly compared to an anti-HA stem bNAb (like CR9114 for example) if the authors suspect Fc-mediated effector functions may be important for protection in the mouse model? It has been repeatedly demonstrated that Fc-mediated functions, like ADCC and ADCP, are required for protection by anti-HA stem bNAbs in vivo. Therefore, an anti-HA stem bNAb should be included as a positive control across all the assays performed instead of OR in addition to F10.

Reply: CR9114 binds and neutralizes a panel of group 1 and group 2 influenza viruses. Like CR9114, F10 is an anti-HA stem bNAb. Both F10 and CR9114 use the V_H1-69 germline segment (PMID: 23583287). We have included F10 as a positive control across all the assays in the original submission, and considered another positive control nNAb with neutralizing ability against HA and similar recognition site as F10 may not be necessary.

3. Previous studies have described broadly binding, non-neutralizing mAbs targeting the globular HA head that are protective in murine models of influenza (DiLillo et al 2016), so this aspect of the study is not particularly novel.

Reply: We thank the reviewer for raising this point. Indeed, Fc-FcγR interactions utilized by non-neutralizing anti-HA head mAbs to mediate protection *in vivo* have been demonstrated, but the underlying immune cells involved in this context were not formally reported. We here provided

evidence that both NK cells and alveolar macrophages are involved for the protective effect of broadly binding, non-neutralizing, mAbs *in vivo*. We have addressed this point and added the reference kindly provided by the reviewer in this revision (lines 253-255, page 15).

4. The most novel aspect of this study, in my opinion, is the NK cell and alveolar macrophage co-depletion data. These co-depletion assays should be performed with other broadly binding HA mAbs (HA stem mAbs and other globular head mAbs with known epitopes) to determine whether NK cells and alveolar macrophages are involved in mediating protection against other HA epitopes OR if this is unique to the epitope recognized by the 651 mAb.
Reply: We thank the reviewer for noting the novelty of our co-depletion study. In fact, in our original submission (Figure 5), we included F10 (a broadly binding and neutralizing mAb targeting to stem region) in our co-depletion assay. Results showed that, up co-depletion of NK cells and alveolar macrophages, F10 remained to effectively protect the mice from the challenge with a lethal dose of influenza virus (Figure 5B), suggesting that the effect of a potent broadly neutralizing mAb *in vivo* may not critically rely on the effector functions, and that NK cells and alveolar macrophages are important for the protective function of non-neutralizing mAb recognizing head region of HA.

5. Why is there a massive decrease in survival for the control liposome + control antibody mice that received 651 (Figure 5A, 3rd panel)? In this group, only 10% of mice that got 651 survived compared to 100% of mice that received F10. Why would the control liposomes + control antibody have this effect on 651 treated mice? This finding is completely ignored in the Results/Discussion and needs to be addressed. It is very strange the control liposomes and control antibody would have this kind of impact on protection by 651 and suggests some kind of experimental issue (possibly with the liposomes).
Reply: We thank the reviewer for noting the unexpected effect of control liposome (Figure 5A) on 651 (1st and 3rd panel) or on F10 (1st panel) treatment. Although have not been formally demonstrated, we suspected that intranasal administration of liposome alone might absorb or affect the function of antibody. We have included this point in this revision (page 14, lines 235-237).

6. What is this control IgG that was used as the negative control in the ADCC and ADCP assays? Some details about this are required to know if this is a suitable negative control, especially since some background killing was observed in ADCC assay (20% in 50ug/ml wells for Bris/07). What was the % cytotoxicity in the no antibody control wells of the cytotoxicity assay?
Reply: We thank the reviewer for carefully reading our manuscript. The control IgG used in ADCC and ADCP was purchased from GeneTex (GTX16193), which is a human IgG1 kappa isotype control mAb. Its information is included in this revision (pages 23 and 24). Some background killing was observed in ADCC (20% in 50 ug/ml wells for Bris/07, but not for Cal/09, H3 and H7, Figure 4C) in our original submission. We believe that this slight high background was from the experimental/individual donor variations, as control IgG at 50 ug/ml showed low background killing toward the Bris/07 expressing cells in Figure 4D. In fact, the statistical analysis of various concentrations of IgG killing of Bris/07 expressing cells did not appear to be significantly different among 50, 25 and 12.5 ug/ml groups (results are attached to the right).

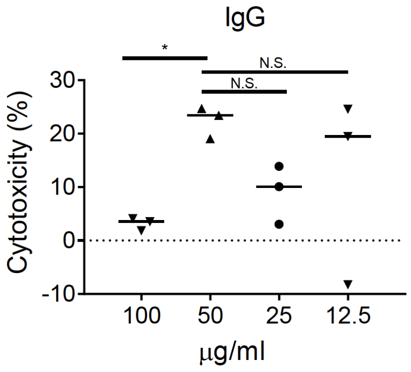

Part III – Minor Issues: Editorial and Data Presentation Modifications
Reviewer #3: Minor issues:
1. Line 47: Influenza D also exists. Reply: We have made the correction.

2. Lines 49-50: Saying that seasonal influenza vaccination protects 2/3rds of people vaccinated is not accurate. This varies tremendously year-to-year depending on how well the vaccine strains of

influenza virus match the strains of influenza virus circulating in the population. Reply: We have made the correction.
