# Supplementary material for: A non-neutralizing antibody broadly protects against influenza virus infection by engaging effector cells
Source: PLoS Pathog. 2021 Aug 5;17(8):e1009724. doi: 10.1371/journal.ppat.1009724 (PMC8341508; doi:10.1371/journal.ppat.1009724)
Supplement: Attachment — Submitted filename: response to reviewer 3s comments.pdf [file ppat.1009724.s007.pdf]

Reviewer #3:

Part II – Major Issues: Key Experiments Required for Acceptance

The authors have not addressed the below experimental concern to my satisfaction:

Why is there a massive decrease in survival for the control liposome + control antibody mice that received 651 (Figure 5A, 3rd panel)? In this group, only 10% of mice that got 651 survived compared to 100% of mice that received F10. Why would the control liposomes + control antibody have this effect on 651 treated mice? This finding is completely ignored in the Results/Discussion and needs to be addressed. It is very strange the control liposomes and control antibody would have this kind of impact on protection by 651 and suggests some kind of experimental issue (possibly with the liposomes).

Reply: We thank the reviewer for noting the unexpected effect of control liposome (Figure 5A) on 651 (1st and 3rd panel) or on F10 (1st panel) treatment. Although have not been formally demonstrated, we suspected that intranasal administration of liposome alone might absorb or affect the function of antibody. We have included this point in this revision (page 14, lines 235-237).

The authors did respond to this comment, but they did NOT address why in the 3rd panel of Figure 5A the liposome + control antibody treatment brought the survival of 651 mAb treated mice down to only 10% while 100% of F10 mAb treated mice survived. If this was a non-specific antibody-liposome interaction as the authors suggest, why wasn't a reduction in survival observed for the F10 treated group as well? I was hopeful that the authors would have either repeated this mouse experiment or provided some kind of logical explanation for this strange result. Since they have done neither of these things, it is difficult to decipher the results of this critical murine co-depletion assay.

The competition ELISA performed with a single HA-head mAb represented the absolute minimum but it was satisfactory, if barely.

Reply:

We thank the reviewer for raising this critical concern. In this revision, we provided more explanation for the reduced survival resulted from the intranasal treatment of liposome + control antibody after influenza virus infection on page 14 (lines 237~244). Administration of the control liposome may not represent the best control for clodronate liposome treatment as liposomes block phagocytosis or affect other macrophage functions (PMID: 8083541). Therefore, sham injection was suggested as the control for mimicking deletion treatments (PMID: 8083541). Supporting this notion, in this revision, our additional data in Supplementary Figure 4A showed that, as compared with sham treatment, control liposome indeed substantially reduced the frequency of alveolar macrophages. However, as there were still some residual alveolar macrophages left in the liposome + control antibody treated mice, about 10% of them were still protected by 651 mAb treatment after influenza virus challenge. Together, we thought that the reduced protective effect of 651 mAb resulting from liposome + control antibody treatment may be due to the reasons that the frequency of alveolar macrophages was dramatically reduced after liposome + control antibody treatment and that ADCC is critical for the effect of 651 mAb. On the other hand, liposome + control antibody treatment did not largely affect the protective efficacy of F10 because the activity of F10 mainly relies on neutralization.
